# Distinct DNA methylation targets by aging and chronic inflammation: a pilot study using gastric mucosa infected with *Helicobacter pylori*

**DOI:** 10.1186/s13148-019-0789-8

**Published:** 2019-12-11

**Authors:** Satoshi Yamashita, Sohachi Nanjo, Emil Rehnberg, Naoko Iida, Hideyuki Takeshima, Takayuki Ando, Takao Maekita, Toshiro Sugiyama, Toshikazu Ushijima

**Affiliations:** 1 Division of Epigenomics, National Cancer Center Research Institute, 5-1-1 Tsukiji, Chuo-ku, Tokyo, 104-0045 Japan; 2 Third Department of Internal Medicine, University of Toyama, Toyama, Japan; 3 Second Department of Internal Medicine, Wakayama Medical University, Wakayama, Japan

**Keywords:** DNA methylation, *Helicobacter pylori*, Aging, CpG island

## Abstract

**Background:**

Aberrant DNA methylation is induced by aging and chronic inflammation in normal tissues. The induction by inflammation is widely recognized as acceleration of age-related methylation. However, few studies addressed target genomic regions and the responsible factors in a genome-wide manner. Here, we analyzed methylation targets by aging and inflammation, taking advantage of the potent methylation induction in human gastric mucosa by *Helicobacter pylori* infection-triggered inflammation.

**Results:**

DNA methylation microarray analysis of 482,421 CpG probes, grouped into 270,249 genomic blocks, revealed that high levels of methylation were induced in 44,461 (16.5%) genomic blocks by inflammation, even after correction of the influence of leukocyte infiltration. A total of 61.8% of the hypermethylation was acceleration of age-related methylation while 21.6% was specific to inflammation. Regions with H3K27me3 were frequently hypermethylated both by aging and inflammation. Basal methylation levels were essential for age-related hypermethylation while even regions with little basal methylation were hypermethylated by inflammation. When limited to promoter CpG islands, being a microRNA gene and high basal methylation levels strongly enhanced hypermethylation while H3K27me3 strongly enhanced inflammation-induced hypermethylation. Inflammation was capable of overriding active transcription. In young gastric mucosae, genes with high expression and frequent mutations in gastric cancers were more frequently methylated than in old ones.

**Conclusions:**

Methylation by inflammation was not simple acceleration of age-related methylation. Targets of aberrant DNA methylation were different between young and old gastric mucosae, and driver genes were preferentially methylated in young gastric mucosa.

## Introduction

Aberrant DNA methylation is deeply involved in various human disorders, including cancers [1]. It is present not only in cancer tissues but also in normal tissues, and its accumulation levels are associated with cancer risk in multiple types of cancers [2, 3]. Such methylation in normal tissues is induced by multiple factors, such as aging and chronic inflammation [4, 5]. Aging has been known to be associated with aberrant DNA methylation for decades [6] and is now believed to induce aberrant DNA methylation as a result of an increased number of cell divisions [7]. At the same time, chronic inflammation, such as gastritis triggered by *Helicobacter pylori* (*HP*) infection, ulcerative colitis, and virus-induced hepatitis, potently increases aberrant DNA methylation in normal tissues [7–10]. The induction mechanism by chronic inflammation has been considered as acceleration of age-related methylation [7, 8].

Nevertheless, target genes for methylation induction in normal tissues by aging and chronic inflammation are still elusive, mainly due to low DNA methylation levels, even if induced. One potentially useful resource is gastric mucosae with chronic inflammation due to *HP* infection that can have high levels of aberrant DNA methylation [11–13] and whose methylation burden can predict cancer risk if appropriately measured [14–17]. By analysis of a small number of promoter CpG islands (CGIs), the presence of target gene specificity of methylation induction by chronic inflammation was suggested [18]. Recently, genome-wide DNA methylation analysis clearly showed that a large number of CpGs were preferentially methylated by *HP* infection-triggered inflammation [19–22]. Especially, Woo et al. showed distinct methylation changes associated with *HP* infection and cancer risk [21].

In contrast with methylation targets in normal tissues, those in cancer cells, which can be readily identified, have been extensively studied, and multiple factors for target determination are known. First, a low transcription level of a gene frequently leads to methylation of its promoter CGIs [23–25]. Second, the presence of trimethylation of histone H3 lysine27 (H3K27me3), a DNA methylation-independent repressive histone modification [26], increases methylation susceptibility [27–30]. Third, the presence of RNA polymerase II confers resistance to methylation induction, independently from transcription and H3K27me3 [25, 31]. Fourth, methylation of a few of a large number of CpG sites within a CGI, namely seeds of methylation, has also been reported to confer susceptibility to aberrant DNA methylation of a CGI [23]. Furthermore, microRNA genes are suggested to be susceptible to methylation induction in cancer tissues [32, 33]. If not limited to promoter CGIs, the importance of methylation of CGI shores in tissue- and cancer-specific methylation of CGI has been suggested [34]. Also, a striking association between nuclear lamina-associated domains (LADs) and long-range hypomethylated domains in tumors were observed [35, 36].

In this study, we address whether inflammation-induced methylation can be fully explained as acceleration of age-related methylation. We also address whether methylation targets in young and old gastric mucosae are the same or not.

## Materials and methods

### Tissue samples and *HP* infection status

*HP* never infected individuals under 40 years (“young”; age = 24, 26, 30, 35; *n* = 4), *HP* currently infected young individuals (age = 22, 25, 29, 38; *n* = 4), *HP* never infected individuals above 60 years (“old”; age = 66, 71, 73, 74; *n* = 4), and *HP* currently infected old individuals (age = 73, 76, 78, 85; *n* = 4) were recruited with written informed consents under approval of the institutional review boards (University of Toyama and Wakayama Medical University). These groups were designated as *HP*(−) young, *HP* current young, *HP*(−) old, and *HP* current old, respectively. *HP* infection status was analyzed by Giemsa staining, urea breath test (Otsuka, Tokushima, Japan), rapid urease test (Otsuka), and serum or urine anti-*HP* IgG antibody test (SRL, Tokyo, Japan). *HP* never infected status was estimated by negative results for anti-*HP* IgG antibody test and one of the other three analyses, and was established by the lack of gastric atrophy (Additional file 1: Table S1). *HP* currently infected status was estimated by a positive result in at least one of the mentioned four analyses, and established by positive results by PCR of genomic DNA of *HP* [9]. Past-infected samples from healthy individuals who experienced the successful eradication of *HP* (*HP* past, age = 52–68; *n* = 12) were obtained and analyzed in our previous study [16]. We excluded gastric mucosa obtained from subjects with any malignancies. Data of non-cancerous gastric mucosa of cancer patients (age = 47–65; *n* = 12) were obtained from our previous publications [16].

All gastric samples were endoscopically biopsied from a fixed position in the antral region (2 cm from the pyloric ring on the lesser curvature) at the occasion of a routine screening of gastric cancers and stored in RNAlater (Thermo Fisher Scientific, MA, USA) at − 80 °C until DNA or RNA extraction. Genomic DNA was extracted by the standard phenol/chloroform method and was quantified using a Quant-iT PicoGreen dsDNA Assay Kit (Life Technologies, Carlsbad, CA). Total RNA was isolated using ISOGEN (Nippon Gene, Tokyo, Japan) and was purified using an RNeasy Mini Kit (Qiagen, Valencia, CA).

### Genome-wide DNA methylation analysis

A genome-wide DNA methylation analysis was performed as described previously [37, 38] using an Infinium HumanMethylation450 BeadChip array (HM450), which covers 482,421 CpG sites and 3091 non-CpG (CpA or CpT) sites (Illumina, San Diego, CA). The HM450 data were submitted to the Gene Expression Omnibus (GEO) database under accession # GSE92863. The HM450 data for past-infected samples were obtained from our previous study [16]. The HM450 data for seven kinds of blood cells (CD4+ T cells, CD8+ T cells, CD56+ NK cells, CD19+ B cells, CD14+ monocytes, neutrophils, and eosinophils, *n* = 6 of each) were obtained from the GEO database (GSE35069) [39]. A methylation level was represented by a *β* value that ranged from 0 (unmethylated) to 1 (fully methylated). A total of 8480 cross-reactive/polymorphic probes identified by Chen et al. [40] were removed from the analysis.

### Estimation of blood cell fraction

The HM450 data of the seven cell populations of blood were grouped into three cell groups (T cells (CD4+ T cells, CD8+ T cells, and CD56+ NK cells), B cells (CD19+ B cells), and monocytes (CD14+ monocytes, neutrophils, and eosinophils) based upon the similarity of methylation profiles [39]. Marker CpG sites for T cell, B cell, monocyte, and pan-blood cells were screened as (1) highly methylated (*β* values ≥ 0.5) in a specific type of blood cell, (2) not methylated (*β* values ≤ 0.1) in the other types of blood cells or in *HP*(−) young gastric mucosae. Consequently, 100 candidate marker CpG sites were isolated. However, many of these CpG sites were methylated by *HP*-triggered inflammation, and the CpG sites were methylated to various degrees among individuals.

To resolve this issue, methylation levels of the 100 candidate marker CpG sites were plotted [*x* = Δ*β* value (blood cell − *HP*(−) young), *y* = Δ*β* value (sample − *HP*(-) young)] (Additional file 1: Figure S1A). In this plot, if a CpG site is not methylated by *HP* infection, its *x* value represents its methylation level in a specific blood cell, and its *y* value represents the product of blood cell fraction and the methylation level. In reality, many of the 100 candidate marker CpG sites were methylated by *HP* infection, and their plot is composed of CpG sites not methylated and methylated by *HP* infection. Therefore, we selected the 20 least methylated CpG sites and obtained their regression line to calculate the fraction of specific types of blood cells.

### Evaluation of methylation levels of specific genomic regions

The 482,421 probes for CpG sites were assembled into 276,831 genomic blocks, collections of probes classified by their locations against transcription start sites (TSSs), and CGIs, using a browser-accessible bioinformatics tool, MACON [41]. During the MACON processing, probes that showed a signal intensity less than 500 were filtered out, and intra-array normalization was performed using a peak-based correction method, BMIQ (Beta MIxture Quantile dilation) [42].

From the 276,831 genomic blocks, 270,249 genomic blocks located on autosomes were used for further analysis. The methylation level of a genomic block was defined as a mean *β* value of the probes in the genomic block and was calculated by MACON. The methylation level was corrected by a blood cell fraction in a sample as follows. Corrected methylation level = (measured methylation level − Bm × Bf − Tm × Tf − Mm × Mf)/Gf (Bm, methylation level in B cells; Bf, fraction of B cells; Tm, methylation level in T cells; Tf, fraction of T cells; Mm, methylation level in monocytes; Mf, fraction of monocytes; Gf, fraction of gastric epithelial cells, calculated = 1 − Bf − Tf − Mf). When a value of the corrected methylation level was larger than 1, it was corrected to be 1. A methylation level for a group of samples was defined as a mean corrected methylation level of the samples in the group. An increase or decrease of methylation levels by a *β* value of 0.1 or more was defined as “hypermethylation” and “hypomethylation,” respectively.

### Targeted deep bisulfite sequencing using a next-generation sequencer

A targeted deep bisulfite sequencing was performed using Ion PGM (Thermo Fisher Scientific) as described previously [43]. Sodium bisulfite-treated DNA was amplified using nine primer sets common to methylated and unmethylated DNA sequences (Additional file 1: Table S2). Five hundred sequence reads randomly selected from obtained reads that covered all of the CpG sites in the reference sequences were used for analysis of methylation patterns.

### Gene transcription analysis by oligonucleotide microarray, and H3K27me3 and LAD information

Transcription microarray analysis was performed by a GeneChip Human Genome U133 Plus 2.0 transcription microarray (Affymetrix, Santa Clara, CA) as described previously [25]. The scanned data were processed using GeneChip operating software (Affymetrix). The signal intensity of each probe was normalized so that the mean of the signal intensities of all the probes on a microarray would be 500. The mean of the signal intensities of all probes for a gene was used as the transcription level of the gene. Genes were classified into expressed (> 125) gene and unexpressed (< 125) gene according to their signal intensities.

The information on H3K27me3 modification in gastric mucosa was obtained from GEO (GSM772969). LADs of Tig3 cells [44] and PrEC cells [36] were obtained from UCSC Genome Browser and GEO (GSM2610545), respectively.

### Gene ontology analysis

Gene ontology (GO) analysis was conducted by GOrilla [45]. The CGI/TSS200 hypermethylated genes from each gene set and all of the genes were loaded to GOrilla. The relative ranking order of biological processes was determined using their FDR *q* values.

### Statistical analysis

Multivariate analysis was conducted by binomial logistic regression using SPSS software version 18 (IBM, Chicago, IL). Transcription levels and basal methylation levels (methylation levels in *HP*(−) young) were categorized into quintiles in the binomial logistic regression analysis. Unsupervised hierarchical clustering analysis was performed using R 3.5.1 with the Heatplus package from Bioconductor. Differential methylation levels (*β* values) between two groups were analyzed using Welch's *t*-test with multiple testing corrections applied using the Benjamini-Hochberg false discovery rate.

## Results

### Correction of contaminating leukocyte fraction enabled clear distinction between currently and past-infected gastric mucosae

**Fig. 1:**
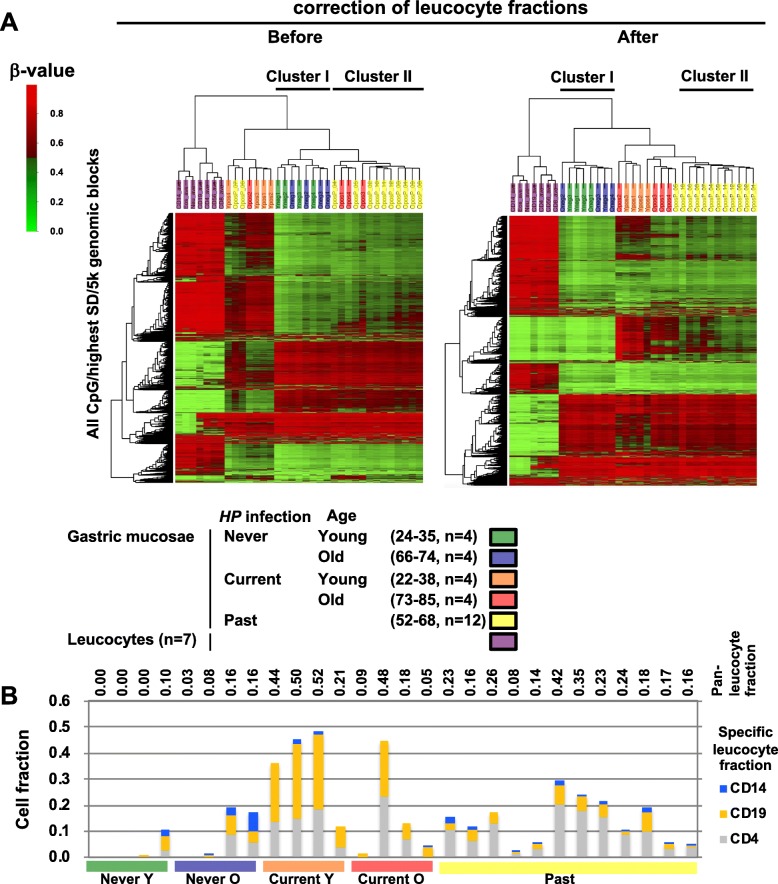
Genome-wide methylation analysis of gastric mucosa and removal of the influence of contaminating leukocytes. **a** Unsupervised hierarchical clustering analysis of methylation levels of gastric mucosa samples and leukocytes. *β* values of top 5000 genomic blocks with the highest standard deviation (SD) among all genomic blocks were used. Before correction of leukocyte fractions, never infected young and old gastric mucosae (cluster I) and past-infected and currently infected old gastric mucosae (cluster II) were mixed up (left). After correction (right), cluster II consisted of only past-infected gastric mucosa samples, and the other clusters also had high accordance with the samples. **b** Fractions of leukocytes in gastric mucosa samples. Fractions of (i) T cells and NK cells (CD4), (ii) B cells (CD19), and (iii) monocytes, neutrophils, and eosinophils (CD14) were separately calculated using specifically methylated CpG sites, and the sum of the three groups was in good accordance with the fraction of pan-leukocytes. A fraction of these cells was used for correction of contaminating leukocytes in gastric mucosa samples

In a genome-wide DNA methylation analysis, large numbers of genomic blocks were hypermethylated (43,698–55,367 of 270,249) and hypomethylated (32,708–55,313) in *HP* currently infected gastric mucosae (*HP* current young), compared with age-matched never infected gastric mucosae [*HP*(−) young]. However, cluster analysis using the 5000 genomic blocks with the highest SD mixed up young and old gastric mucosae (cluster I) and also past-infected (*HP* past) and *HP* current gastric mucosae (cluster II) (Fig. 1a left). This relatively unclear separation suggested that a fraction of methylation changes by *HP* infection, especially in currently infected samples, was due to contamination of other types of cells, namely infiltrating leukocytes.

To estimate the fraction of contaminating leukocytes, we analyzed methylation levels of CpG sites unmethylated in the *HP*(−) group and specifically methylated in leukocytes (Additional file 1: Figure S1A). It was reported that leukocytes can be classified into three groups by the methylation patterns: (i) T cells and NK cells, (ii) B cells, and (iii) monocytes, neutrophils, and eosinophils [39]. Here, the fractions of these three groups were separately calculated using 20 CpG sites highly methylated in respective leukocyte groups but not in gastric mucosa without *HP* infection (Additional file 1: Figure S1B). The methylation levels in gastric epithelial cells were calculated using the fractions of the three groups of leukocytes. The sum of the fraction of the three groups was close to the fraction of pan-leukocytes (Fig. 1b). *HP* current samples showed a high B cell fraction while *HP* past samples showed a high T cell fraction. After correction of the contamination of leukocytes, distinction between currently and past-infected gastric mucosae became clearer (Fig. 1a right). This showed that the influence of contaminating leukocytes in gastric mucosa was successfully removed.

### Inflammation-induced methylation is not simple acceleration of age-related methylation

First, the similarity of methylated genomic blocks among the gastric mucosa samples of four individuals within a group was analyzed for four groups (young and old *HP*(−), and young and old *HP* current groups). Intragroup correlation coefficients of methylation levels were very high (> 0.96), except for *HP* current old #2 sample (Additional file 1: Figure S2). Intergroup correlation was very high between *HP*(−) young and *HP*(−) old groups, and lower between the other groups. Then, aberrant DNA methylation in *HP* current young individuals compared with that in the *HP*(−) young group was analyzed. Sixty-five percent (= 32,814/50,817) or more of genomic blocks hypermethylated (Δ*β* ≥ 0.1) in one individual were commonly hypermethylated in the other three individuals and 60% (= 35,210/59,045) or more of the genomic blocks hypomethylated (Δ*β* ≤ − 0.1) in one individual were commonly hypomethylated in the other three individuals (Additional file 1: Figure S3A). When limited to CGIs, genomic blocks hypermethylated by inflammation were more closely shared by the four individuals in the *HP* current young group, and those hypomethylated were more weakly shared (Additional file 1: Figure S3B). These results showed that chronic inflammation triggered by *HP* infection induced hyper- and hypomethylation in consistent genomic regions, depending upon genomic structure. Therefore, for a genomic block, a mean *β* value of four samples in a group was used as the methylation level of the group. Between males and females, very limited differences were observed using *HP* past samples (male = 7, female = 5; Additional file 1: Figure S4A), and gender composition was considered to have a limited influence.

**Fig. 2:**
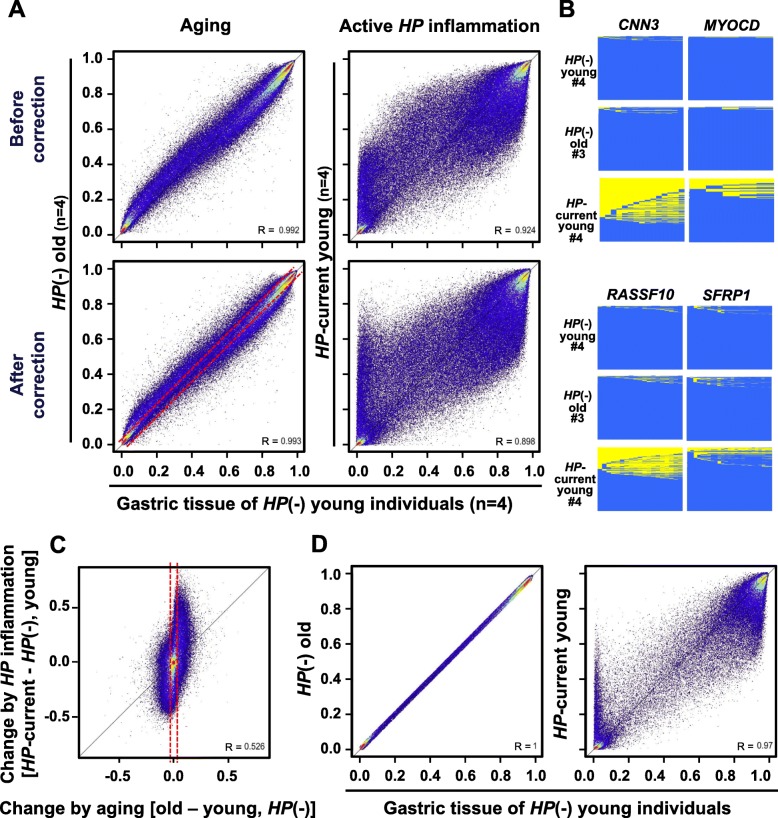
Aberrant DNA methylation induced in gastric mucosae by aging and active inflammation due to current infection. **a** Global difference of methylation levels (*β* values) between *HP*-negative young gastric mucosae (*HP*(−) young) and *HP*-negative old mucosae (*HP*(−) old) and between *HP*-negative young mucosae (*HP*(−) young) and *HP*-positive young mucosae (*HP* current young). Even after correction of contamination of leukocytes, a large number of blocks (44,461 and 51,078 blocks (16.5% and 18.9%)) were aberrantly hyper- or hypomethylated by inflammation. **b** Targeted deep bisulfite sequencing for the CGI/TSS200 regions of four genes. Yellow square, methylated CpG site; blue square, unmethylated CpG site. **c** Methylation changes by aging and inflammation. The horizontal axis shows methylation changes by aging and the vertical axis shows methylation changes by inflammation. Most genomic blocks hyper- or hypomethylated by inflammation were also hyper- or hypomethylated at low levels by aging. **d** Methylation changes by inflammation in the 141,892 blocks not methylated by aging (Δ*β* value < 0.02, inside dotted line in (**c**)). 9596 and 17,450 blocks (6.8% and 12.3%) were hyper- or hypomethylated in *HP* current young gastric mucosae

Genomic blocks methylated by aging were identified by comparing the *HP*(−) old gastric mucosa with the *HP*(−) young gastric mucosa. A total of 7315 (2.7%) and 5445 (2.0%) genomic blocks were hyper- and hypomethylated, respectively (Additional file 1: Figure S4B; Table S3). Genomic blocks methylated by *HP*-triggered inflammation were also identified by comparing the *HP* current young gastric mucosa with the *HP*(−) young gastric mucosa. A total of 44,461 (16.5%) and 51,078 (18.9%) genomic blocks were hyper- and hypomethylated, respectively (Additional file 1: Figure S4C; Table S3). In addition to the large numbers, the levels of aberrant DNA methylation induced by inflammation were markedly higher than those by aging, even after correction of the leukocyte fraction (Fig. 2a). Targeted deep bisulfite sequencing was performed for genes randomly selected from genes whose CGI/TSS200 were hyper- or not methylated by *HP* infection, and induction of methylation of multiple CpG sites within a DNA molecule by inflammation was confirmed (Fig. 2b; Additional file 1: Figure S5).

Differences between current and past infection were also analyzed. Methylation induction in *HP* current old and *HP* past (old) gastric mucosae was assessed by comparing their *β* values with those in *HP*(−) old gastric mucosa (Additional file 1: Figure S4D; Figure S6). A total of 39,312 (14.5%) and 39,276 (14.5%) genomic blocks were hyper- and hypomethylated, respectively, by current infection. In contrast, smaller numbers of genomic blocks (28,861 (10.7%) and 14,344 (5.3%)) were hyper- and hypomethylated, respectively, by past infection, and the degrees of hyper- and hypomethylation were smaller. This was in line with our and others’ previous findings that more extensive aberrant DNA methylation is present in currently infected gastric mucosa [12, 46, 47].

Overlapping of genomic blocks hyper- (or hypo-) methylated by aging and inflammation was then examined. The degree of changes by inflammation was compared with that by aging, and most blocks hyper- (or hypo-) methylated by inflammation were found to be also hyper- (or hypo-) methylated at low levels by aging (Fig. 2c). At the same time, even when genomic blocks were limited to 141,892 genomic blocks unaffected by aging, 9596 and 17,450 blocks (6.8% and 12.3%) were hyper- or hypomethylated in *HP* current young gastric mucosae (Fig. 2d). These results showed the presence of specific targets of aberrant DNA methylation by *HP*-triggered inflammation.

In addition, methylation of known driver genes and cancer risk marker genes [15, 16] was analyzed. Driver genes were slightly methylated by aging, and more extensively methylated by inflammation. Risk marker genes also showed similar methylation profiles (Additional file 1: Figure S7A). In contrast, methylation levels of driver genes were similar between non-cancerous gastric mucosa of cancer patients and *HP* past healthy people, but those of cancer risk marker genes were different (Additional file 1: Figure S7B).

### Genomic factors differentially influence hyper- and hypomethylation by aging and inflammation

First, genomic blocks were classified according to their methylation dynamics by aging and inflammation. For this, all genomic blocks were plotted with the methylation changes by aging in the *x*-axis, and those by *HP*-triggered inflammation in the *y*-axis (Additional file 1: Figure S8). Blocks hyper- and hypomethylated only by inflammation (those in Fig. 2d) were plotted in areas C1 and F1 (inflammation-specific hyper- and hypomethylation, respectively). Blocks hyper- and hypomethylated by aging and markedly accelerated by inflammation were plotted in areas B1 and E1, respectively (inflammation-accelerated hyper- and hypomethylation, respectively). Blocks hyper- and hypomethylated only by aging were plotted in areas A1 and D1 (aging-specific hyper- and hypomethylation, respectively). Although the “aging-specific” genomic blocks could contain those weakly methylated by inflammation, the numbers of such blocks were still small. Among the 44,461 genomic blocks hypermethylated by inflammation, 9596 (21.6%) and 27,491 (61.8%) were inflammation specific and inflammation accelerated, respectively (Additional file 1: Table S3). Among the 51,078 genomic blocks hypomethylated, 17,450 (34.2%) and 26,202 (51.3%) were inflammation specific and inflammation accelerated, respectively.

The influences of locations against a CGI and a gene on hypermethylation were then examined. Regarding the influence of a CGI, inflammation-specific and inflammation-accelerated hypermethylation were induced frequently in CGIs (Additional file 1: Figure S9A). However, when the analysis was limited to TSS200 regions, the influence of CGIs became unclear (Additional file 1: Figure S9B). Regarding the influence of locations against a gene, inflammation-accelerated methylation was frequently observed in the 1st exons (Additional file 1: Figure S9C). When limited to CGIs, the influence of the 1st exon became weaker than that of intergenic regions (Additional file 1: Figure S9D).

Third, the influences of the locations on hypomethylation were examined. Regarding the influence of a CGI (Additional file 1: Figure S10A, B), hypomethylation was induced infrequently in CGIs, even when limited to TSS200. Regarding the influence of locations against a gene (Additional file 1: Figure S10C, D), hypomethylation was infrequent in TSS200 regions.

### Basal methylation levels and H3K27me3 also differentially influence hyper- and hypomethylation by aging and inflammation

**Fig. 3:**
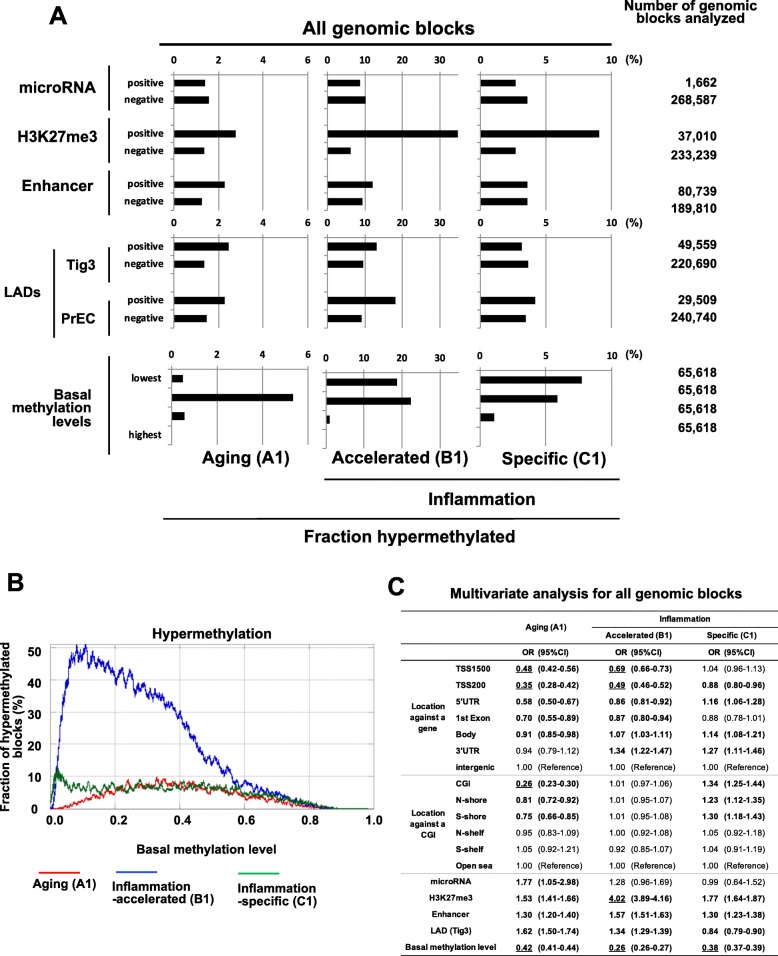
Influence of microRNA, H3K27me3, enhancers, LADs, and basal methylation levels, in addition to genomic factors on hypermethylation. **a** Univariate analysis. The influence of each factor on hypermethylation was estimated by the fraction of hypermethylated genomic blocks. **b** Influence of basal methylation levels on hypermethylation. The fraction of hypermethylated genomic blocks among the blocks with the same basal methylation levels in *HP*(−) young gastric mucosa was plotted. Basal methylation levels larger than 0.1 of *β* value were essential for hypermethylation by aging. In contrast, hypermethylation by inflammation was induced even in genomic blocks with basal methylation levels smaller than 0.1 and was very frequent with basal methylation levels between 0.1 and 0.5. **c** Multivariate analysis involving locations against a gene and a CGI, microRNA, H3K27me3, enhancers, LADs, and basal expression levels. Boldface; statistically significant, underline; odds ratio > 2.0 or < 0.5

In addition to genomic factors, we further analyzed factors known to be related to aberrant DNA hypermethylation, including (i) being a microRNA gene (microRNA), (ii) the presence of H3K27me3 modification (H3K27me3), (iii) being in an enhancer region (enhancers), (iv) being in LADs (LADs), and (v) having a high basal methylation level. In the total genomic regions, the influence of microRNA genes was not observed for hypermethylation by aging or inflammation (Fig. 3a). Genes with H3K27me3 in *HP*(−) young gastric mucosa were frequently hypermethylated both by aging and inflammation. Enhancers and LADs were frequently hypermethylated by aging. Notably, a basal methylation level (*β* ≥ 0.1 in *HP*(−) young) was essential for hypermethylation by aging, while even regions with very low methylation levels (0.1 > *β* > 0.01) could be methylated by inflammation (Fig. 3b). A multivariate analysis taking account of the genomic structures and these additional factors showed that hypermethylation by aging was strongly influenced by the location against a gene, the location against a CGI, and the basal methylation level (Fig. 3c). On the other hand, hypermethylation by inflammation was strongly influenced only by the basal methylation level. CGIs were unlikely to be hypermethylated by aging but likely to be by inflammation.

Regarding hypomethylation, the absence of H3K27me3 and enhancers was associated with frequent hypomethylation by aging and inflammation (Additional file 1: Figure S11A). By inflammation, aberrant hypomethylation was induced even in regions with very high methylation levels (*β* ≥ 0.9; Additional file 1: Figure S11B). Multivariate analysis showed that only CGIs were extremely unlikely to be hypomethylated either by aging or by inflammation (Additional file 1: Figure S11C). LADs and regions with high basal methylation levels were unlikely to be hypomethylated by aging but likely to be by inflammation. Enhancers were likely to be hypomethylated both by aging and inflammation.

### Promoter CGI hypermethylation by aging and inflammation is influenced by the basal methylation level and H3K27me3, respectively

**Fig. 4:**
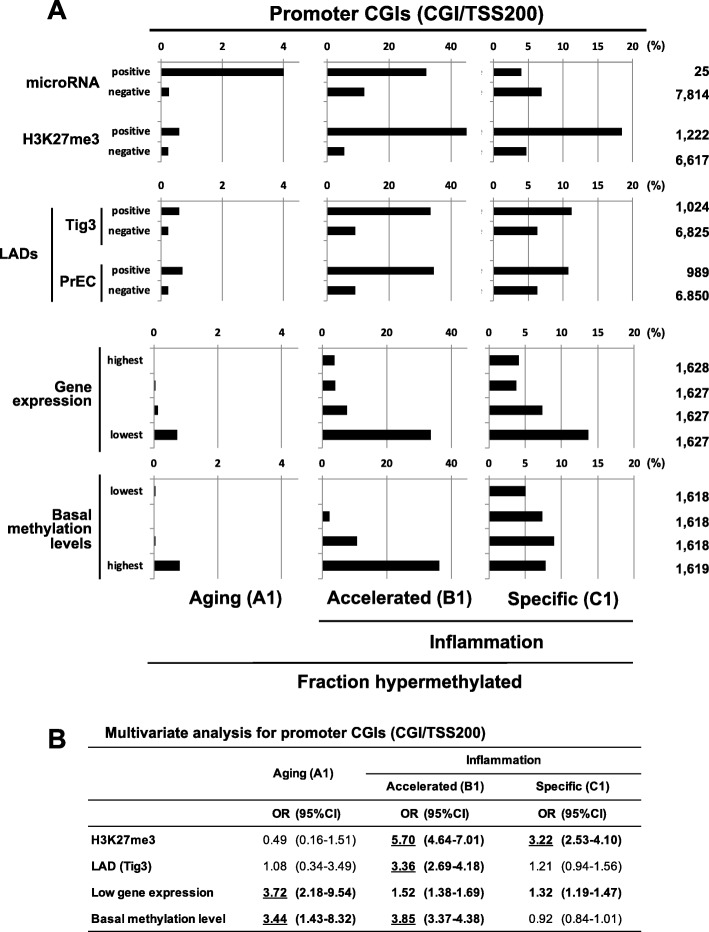
Influences of microRNA genes, H3K27me3, LADs, gene expression levels, and basal methylation levels on hypermethylation of promoter CGIs (CGI/TSS200). **a** Univariate analysis. **b** Multivariate analysis involving H3K27me3, LADs, gene expression levels, and basal methylation levels. MicroRNA was not included because of its small number. A total of 6473 genes with information on the four variates were used

Promoter CGIs are especially important since their methylation silences their downstream genes [48]. As an inducer of their methylation, low expression levels of their downstream genes are known [23–25]. Therefore, we examined the influences of genomic factors, microRNA genes, H3K27me3, enhancer, LADs, basal methylation level, and gene expression level on hypermethylation of promoter CGIs (CGI/TSS200). MicroRNA genes were frequently hypermethylated by aging. Genes with H3K27me3 and those in LADs were frequently hypermethylated by inflammation (Fig. 4a). Genes with low transcription levels were frequently hypermethylated both by aging and inflammation. Notably, genes with high expression were rarely methylated by aging, but even these genes could be methylated by inflammation. Only genes with high basal methylation levels were hypermethylated by aging while the levels did not influence inflammation-induced hypermethylation.

Gene ontology analysis was conducted, but enrichment of cancer-related processes was unclear for genes hypermethylated by aging (A1) or inflammation (C1) (Additional file 1: Table S4). A multivariate analysis taking account of H3K27me3, LADs, gene expression, and the basal methylation level was also performed (Fig. 4b). Although the number of aging-specific hypermethylated genes was only 22 (Additional file 1: Table S3), the influence of low gene expression and the basal methylation level on hypermethylation was clearly observed. In contrast, hypermethylation by inflammation was strongly enhanced by the presence of H3K27me3.

### Driver genes are targeted preferentially in young gastric mucosa

**Fig. 5:**
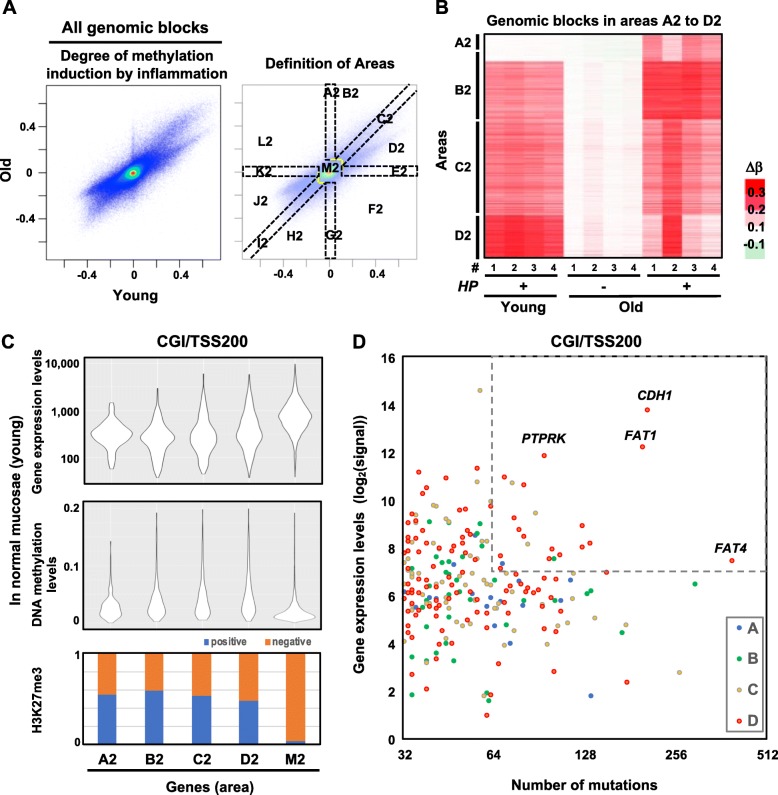
Difference in target genes by inflammation-induced hypermethylation between young and old gastric mucosae, and enrichment of driver genes in young mucosae. **a** Plot of the induction levels in young and old gastric mucosae. A total of 13 areas (A2 to M2) were defined based upon the degrees in young and old gastric mucosae. Genomic blocks in area M2 were not methylated by inflammation in young or old gastric mucosae (Δ*β* values < 0.1). **b** Heatmap of hypermethylated genomic blocks for three kinds of gastric mucosae. The regions in area D2 were hypermethylated in all HP current young gastric mucosae and one or two of the HP current old gastric mucosae. **c** Gene expression levels, basal methylation levels, and H3K27me3 frequency for genes in areas A2-D2 and M2. Regions in area D2 had higher expression levels and lower H3K27me3 frequency. **d** Number of somatic mutations in gastric cancers registered at the COSMIC database and gene expression levels for genes in areas A2-D2. Genes in area D2 were enriched as those with a large number of mutations and high expression levels

The fraction of leukocytes in gastric mucosae was larger in young gastric mucosae than in old ones (Fig. 1b), and this may result in different targets for hypermethylation in young and old gastric mucosae. To address this issue, all the genomic blocks were plotted with the degree of methylation induction in young gastric mucosae in the *x*-axis and that in old gastric mucosae in the *y*-axis. Genomic blocks hyper- and hypomethylated equally in young and old mucosae were plotted in areas C2 and I2, respectively (Fig. 5a). Genomic blocks that were hyper- and hypomethylated more in old mucosae than in young ones were plotted in areas B2 and H2, respectively. Small numbers of genomic blocks were specifically hypermethylated (area A2) and hypomethylated (area G2) in old gastric mucosae. A large number of genomic blocks appeared to be hyper- (area D2) and hypomethylated (area J2) more in young gastric mucosae. A heatmap of the genomic blocks in area A2, B2, C2, and D2 showed that genomic blocks in area D2 were hypermethylated in all of the young gastric mucosae samples and one of the old gastric mucosa samples (#2, Fig. 5b).

When limited to CGI/TSS200, genes more methylated in young gastric mucosae had higher expression levels and a relatively lower frequency of H3K27me3 (Fig. 5c). Analysis of our previous data of 50 primary gastric cancers and 28 gastric cancer cell lines showed that methylation frequency was especially low for CGI/TSS200 in area D2 (Additional file 1: Figure S12) [49]. Driver genes in gastric cancer, such as *CDH1* and *FAT4*, were present in area D2, and genes involved in cell adhesion were enriched (Additional file 1: Table S5). To analyze whether driver genes were enriched in area D2, we plotted the number of mutations in gastric cancer tissues in the COSMIC database and the expression level in gastric mucosae for individual genes in areas A2-D2. Genes in area D2 had large numbers of mutations and higher expression levels than those in other areas (Fig. 5d; Additional file 1: Table S6). This result showed that driver genes were likely to be enriched in area D2 and were preferentially targeted for aberrant hypermethylation in young gastric mucosae.

## Discussion

A large number of CpG sites were strongly hypermethylated by inflammation, and 61.8% of the aberrant hypermethylation was acceleration of low-level methylation by aging. At the same time, 21.6% of the CpG sites were methylated specifically by inflammation and not simple acceleration of age-related methylation. When factors that influence hypermethylated genomic regions were compared between aging and inflammation, most factors commonly influenced hypermethylation, suggesting that common mechanisms were working for methylation induction by aging and inflammation. At the same time, our multivariate analysis revealed that CGIs were likely to be hypermethylated by inflammation but unlikely to be by aging. The finding is consistent with Woo’s first finding that most hypermethylated probes in *HP*-infected gastric mucosae were located within CpG islands [21]. When limited to promoter CGIs (CGI/TSS200), the presence of H3K27me3 strongly enhanced hypermethylation by inflammation while low transcription levels and low basal methylation levels were essential for hypermethylation by aging. The low basal methylation level corresponds to sparse methylation of a CGI, namely seeds of methylation, and its essential role in methylation induction by aging suggested that age-related methylation may involve low-frequency erroneous methylation of neighboring CpG sites upon enormous cycles of maintenance methylation in life.

Targets of aberrant DNA methylation were different between young and old gastric mucosae with current *HP* infection (active inflammation). Genes with high expression are resistant to methylation induction, and their methylation is known as outliers and to contain driver genes [50]. Here, such genes with frequent somatic mutations were hypermethylated preferentially in young gastric mucosae. It was suggested that inflammation in young gastric mucosae is capable of inducing aberrant methylation even in outliers. A potential clue is the difference in the quality and quantity of inflammation in young and old gastric tissues. Young gastric mucosa samples had more infiltration of leukocytes than old ones, and some of them had monocyte (CD14-positive cell) infiltration. This could lead to high expression of *TNF* and *NOS2*, which are considered to be important for methylation induction [9, 51]. The strong induction of methylation and the character of genes methylated, such as cell adhesion, in young gastric mucosae can be an associated specific characteristic of gastric cancer in young patients, such as a high incidence of undifferentiated-type gastric cancer [52–54].

Although driver genes were preferentially hypermethylated in young gastric mucosa, the number of methylated CpG sites and overall methylation levels decreased in gastric mucosae with past *H. pylori* infection (Additional file 1: Figure S6). This indicated that the majority of them were induced in cells other than stem cells, namely in progenitor and differentiated cells. To note, even if a driver gene is methylated in a stem cell, a sufficient number of driver alterations is necessary for cancer development. Therefore, the finding that driver genes were preferentially hypermethylated in young gastric mucosae appears to be consistent with the fact that the incidence of gastric cancer is age dependent.

There are, however, limitations to our study. First, the small number of samples limited the power of statistical methods in the identification of differentially methylated regions. The volcano plot for the effect of aging (Additional file 1: Figure S4B) identified only five hypermethylated and no hypomethylated genomic regions, even with a relaxed significance cutoff value (*q* = 0.1). Such a low number of regions were not concordant with the established knowledge of methylation induction by aging, and introduction of a cutoff value for statistical significance was not possible. Second, the small number of samples did not allow sufficient analysis of variability among human subjects. Although we confirmed high similarity within a group (Additional file 1: Figure S2), one “exception” (*HP* current old #2) was found. Whether the case was a real “exception” or a small group could not be concluded. Third, the small number of samples caused gender mismatch, even though we observed very limited sex differences in the *HP* past samples (Additional file 1: Figure S4A). Lastly, past-infected samples from young healthy individuals could not be analyzed because such samples are rare and difficult to obtain.

We subtracted the influence of DNA methylation of infiltrating leukocytes based upon methylation of CpG sites that were highly methylated in leukocytes and unmethylated in young *HP*(−) gastric mucosae. Since some of this group of CpG sites was methylated in gastric epithelial cells by *HP*-triggered inflammation, we used the 20 least methylated CpG sites. The subtraction enabled us to remove overestimation of aberrant methylation levels in *HP*-positive gastric epithelial cells. Even after the removal, we still detected a large number of genomic blocks hypermethylated or hypomethylated in gastric tissues with inflammation, and the methylation alterations were considered to be in gastric epithelial cells. The method can be applied to methylation analyses of other tissues with infiltrating leukocytes.

We also showed that microRNA genes were susceptible to aberrant methylation by aging. Transcription of microRNA genes can be altered by DNA methylation, as coding genes [55]. At the same time, specific microRNA genes have been suggested to be highly susceptible to aberrant hypermethylation [32, 33]. Our results here supported the previous fragmental findings at a genome-wide level. The independence of the high susceptibility of microRNA genes from other factors was also shown by the multivariate analysis.

## Conclusions

A large number of CpG sites were strongly hyper- and hypomethylated by inflammation. Some of them were specific to inflammation, and methylation by inflammation was not simple acceleration of age-related methylation. In addition, targets of aberrant DNA methylation were different between young and old gastric mucosae, and driver genes were preferentially methylated in young gastric mucosa.

## Supplementary information

**Additional file 1: Table S1–S6**. Supplementary tables. **Figures S1–S12**. Supplementary figures.

## Data Availability

The datasets used in this study are available in the National Center for Biotechnology Information (NCBI) website’s Gene Expression Omnibus (GEO) at https://www.ncbi.nlm.nih.gov/geo/browse/ corresponding to the following GEO Accession number: GSE92863.

## Abbreviations

*HP*: *Helicobacter pylori*
CGI: CpG island
TSS: Transcriptional start site
LAD: Lamina-associated domain
