# Supplementary material for: Distinct DNA methylation targets by aging and chronic inflammation: a pilot study using gastric mucosa infected with Helicobacter pylori
Source: Clin Epigenetics. 2019 Dec 11;11:191. doi: 10.1186/s13148-019-0789-8 (PMC6907118; doi:10.1186/s13148-019-0789-8)
Supplement: Supplementary file 1 — Additional file 1: Table S1–S6. Supplementary tables. Figures S1–S12. Supplementary figures. [file 13148_2019_789_MOESM1_ESM.pdf]

Supplementary Table 1. Characteristics of gastric mucosa samples

Sample name	ID	Age	Gender	Overall *HP* infection status	Giemsa staining test	Urea breath test	Rapid urease test	anti-*HP* IgG antibody		PCR of *HP* genomic DNA	Endoscopic finding of atrophic gastritis
								Serum	Urine		
**Never infected group, *HP* (-)**											
Yneg1	Y-1	24	M	never	nt	-	-	nt	-	-	-
Yneg2	Y-2	26	M	never	nt	-	-	nt	-	-	-
Yneg3	Y-3	30	M	never	-	-	-	nt	-	-	-
Yneg4	Y-4	35	M	never	-	-	-	nt	-	-	-
Oneg1	O-1	71	M	never	nt	-	nt	-	nt	-	-
Oneg2	O-2	74	F	never	nt	-	nt	-	nt	-	-
Oneg3	O-3	66	M	never	nt	nt	-	nt	-	-	-
Oneg4	O-4	73	M	never	-	nt	-	-	nt	-	-
**Current infected group, *HP* current**											
Ypos1	Y+1	22	F	current	nt	+	nt	nt	-	+	closed type
Ypos2	Y+2	25	M	current	nt	nt	nt	+	nt	+	open type
Ypos3	Y+3	29	F	current	nt	nt	nt	+	nt	+	- †
Ypos4	Y+4	38	M	current	nt	nt	nt	+	nt	+	closed type
Opos1	O+1	73	M	current	nt	nt	nt	+	nt	+	open type
Opos2	O+2	76	F	current	nt	nt	nt	+	nt	+	closed type
Opos3	O+3	78	F	current	nt	nt	nt	+	nt	+	open type
Opos4	O+4	85	M	current	nt	nt	nt	+	nt	+	open type

M, male; F, female; nt, not tested
† nodular gastritis.

Supplementary Table 2. PCR primers for bisulfite sequencing

Gene symbol	Forward primer (5' -> 3')	Reverse primer (5' -> 3')	Product length (bp)
ACHE	gtttgtgtttgttattgtatgagg	ccaaacgcactccaataccaa	168
ACTB	gaaagttgttttttatggtt	actaaccaaacttacctaa	163
CNN3	agagtttgggaggaggtaga	cccaaccacaaaccattcac	137
GAPDH	gtgtgtgtttagttgaattag	aaccaatcccaacccaaaatc	124
MYOCD	gattggttgagagtttgttagtagtaaag	catcccaaaccttcctatctaat	126
RASSF10	ttgtygtgaaatatatgaatttatt	taaacctccaaacaactcccaaa	166
RPA2	agtttttgaggtgtgtaattt	cttctaccatcacttcctttat	150
SFRP1	ttgttttttaaggggtgttgag	ctccaaaaactacaaaactaaaatac	199
ST3GAL6	taataaagtagggttttgtttatggagaga	caaactctacccttcaaaaacaaatat	137

All regions sequenced were located in promoter CpG islands (CGI/TSS200).

Supplementary Table 3. Number of genomic blocks classified by sensitivity to aging and/or inflammation

Group*	Number of genomic blocks					
	All genomic regions			CGI/TSS200		
Hypermethylation by aging (Δβ value ≥0.1)	7,315	2.7%	100%	132	1.7%	100%
A1 (Aging-specific hypermethylation)	4,214		57.6%	22		16.7%
Others	3,101		42.4%	110		83.3%
Hypermethylation by inflammation (Δβ value ≥0.1)	44,461	16.5%	100%	1,535	19.6%	100%
B1 (Inflammation-accelerated hypermethylation)	27,491		61.8%	952		62.0%
C1 (Inflammation-specific hypermethylation)	9,596		21.6%	541		35.2%
Others	7,374		16.6%	42		2.7%
Hypomethylation by aging (Δβ value ≤-0.1)	5,445	2.0%	100%	8	0.1%	100%
D1 (Aging-specific hypomethylation)	3,956		72.7%	8		100%
Others	1,489		27.3%	0		0.0%
Hypomethylation by inflammation (Δβ value ≤-0.1)	51,078	18.9%	100%	11	0.1%	100%
E1 (Inflammation-accelerated hypomethylation)	26,202		51.3%	0		0.0%
F1 (Inflammation-specific hypomethylation)	17,450		34.2%	6		54.5%
Others	7,426		14.5%	5		45.5%
Number of all of the genomic blocks analyzed	270,249	100%		7,839	100%	

*Classified according to the criteria in Supplementary Fig. 8

Supplementary Table 4. Gene ontology analysis of promoter CGIs hypermethylated by aging and/or inflammation

Gene group *	GO term	Description	*P* value †	FDR *q* value	Enrichment
A1	GO:0022412	cellular process involved in reproduction in multicellular organism	2.0E-04	1.0E+00	3.71
(n =15)	GO:0001675	acrosome assembly	3.4E-04	1.0E+00	3.47
B1	GO:0032501	multicellular organismal process	1.5E-21	1.9E-17	1.72
(n=803)	GO:0007186	G protein-coupled receptor signaling pathway	6.4E-21	4.1E-17	2.84
	GO:0009653	anatomical structure morphogenesis	6.5E-21	2.8E-17	2.06
	GO:0048856	anatomical structure development	1.9E-19	5.9E-16	1.63
	GO:0032502	developmental process	3.1E-19	8.0E-16	1.47
	GO:0003008	system process	3.0E-17	6.3E-14	2.18
	GO:0050877	nervous system process	7.8E-17	1.4E-13	2.51
	GO:0023052	signaling	1.5E-16	2.3E-13	2.73
	GO:0007267	cell-cell signaling	2.8E-16	3.9E-13	2.91
	GO:0007155	cell adhesion	1.4E-15	1.8E-12	2.42
C1	GO:0032501	multicellular organismal process	9.4E-09	1.2E-04	1.58
(n=472)	GO:0044057	regulation of system process	1.9E-08	1.2E-04	2.56
	GO:0009653	anatomical structure morphogenesis	2.3E-08	9.6E-05	1.83
	GO:0048646	anatomical structure formation involved in morphogenesis	1.1E-07	3.6E-04	2.15
	GO:1903522	regulation of blood circulation	6.9E-07	1.8E-03	3.02
	GO:0023052	signaling	8.3E-07	1.8E-03	2.36
	GO:0001525	angiogenesis	8.7E-07	1.6E-03	2.91
	GO:0007165	signal transduction	8.9E-07	1.4E-03	1.39
	GO:0003008	system process	1.2E-06	1.7E-03	1.88
	GO:0032879	regulation of localization	1.5E-06	2.0E-03	1.49

* Classified according to the criteria in Supplementary Fig. 8

† less than 0.001, top10 GO term

Supplementary Table 5. Gene ontology analysis of promoter CGIs hypermethylated in young and/or old gastric mucosae

Gene group *	GO term	Description	*P* value †	FDR *q* value	Enrichment
A2	GO:0006813	potassium ion transport	1.1E-04	3.4E-01	6.29
(n =120)	GO:0071804	cellular potassium ion transport	8.6E-05	3.6E-01	6.51
	GO:0071805	potassium ion transmembrane transport	8.6E-05	5.4E-01	6.51
	GO:0048853	forebrain morphogenesis	6.4E-05	8.0E-01	31.81
	GO:0061339	establishment or maintenance of monopolar cell polarity	9.7E-04	8.1E-01	14.46
	GO:0003323	type B pancreatic cell development	3.4E-04	8.6E-01	19.88
	GO:0043030	regulation of macrophage activation	9.7E-04	8.7E-01	14.46
	GO:0002068	glandular epithelial cell development	5.1E-04	9.1E-01	17.67
	GO:0022612	gland morphogenesis	9.7E-04	9.4E-01	14.46
	GO:0030001	metal ion transport	9.1E-04	9.6E-01	2.87
B2	GO:0007218	neuropeptide signaling pathway	2.0E-08	2.5E-04	7.60
(n=279)	GO:0007186	G protein-coupled receptor signaling pathway	6.0E-07	3.8E-03	2.73
	GO:0051963	regulation of synapse assembly	4.5E-06	1.9E-02	6.62
	GO:0043269	regulation of ion transport	2.9E-05	9.3E-02	2.38
	GO:0003008	system process	6.4E-05	1.6E-01	1.96
	GO:0048856	anatomical structure development	6.6E-05	1.4E-01	1.48
	GO:0032501	multicellular organismal process	9.4E-05	1.7E-01	1.51
	GO:0034765	regulation of ion transmembrane transport	9.9E-05	1.6E-01	2.55
	GO:0007267	cell-cell signaling	9.9E-05	1.4E-01	2.55
	GO:0042391	regulation of membrane potential	1.6E-04	2.0E-01	2.61
C2	GO:0032501	multicellular organismal process	6.7E-11	8.4E-07	1.75
(n=376)	GO:0009653	anatomical structure morphogenesis	1.2E-10	7.4E-07	2.12
	GO:0023052	signaling	3.4E-10	1.4E-06	3.05
	GO:0007267	cell-cell signaling	7.2E-10	2.3E-06	3.21
	GO:0007186	G protein-coupled receptor signaling pathway	1.6E-09	4.1E-06	2.80
	GO:0003002	regionalization	3.7E-09	7.7E-06	3.70
	GO:0032502	developmental process	6.7E-09	1.2E-05	1.46
	GO:0003008	system process	2.3E-08	3.6E-05	2.18
	GO:0050806	positive regulation of synaptic transmission	3.4E-08	4.7E-05	4.79
	GO:0048598	embryonic morphogenesis	7.0E-08	8.8E-05	2.87
D2	GO:0032501	multicellular organismal process	5.7E-16	7.2E-12	1.67
(n=680)	GO:0009653	anatomical structure morphogenesis	6.5E-15	4.1E-11	1.97
	GO:0007155	cell adhesion	1.2E-14	4.9E-11	2.51
	GO:0022610	biological adhesion	2.2E-14	6.8E-11	2.48
	GO:0048856	anatomical structure development	7.6E-14	1.9E-10	1.57
	GO:0032502	developmental process	3.0E-13	6.4E-10	1.42
	GO:0007389	pattern specification process	1.1E-10	2.0E-07	2.60
	GO:0048729	tissue morphogenesis	1.0E-09	1.6E-06	2.51
	GO:0007186	G protein-coupled receptor signaling pathway	1.3E-09	1.8E-06	2.24
	GO:0040011	locomotion	4.1E-09	5.2E-06	1.93

* Classified according to the criteria in Fig. 5A

† less than 0.001, top10 GO term

Supplementary Table 6. Methylation levels of 30 genes with mutations in gastric cancers

Region ID	Chr	Gene name	Number of mutations in gastric cancers †	Expression in *HP* (-) gastric mucosae (young)	Number of references in PubMed	Methylation level (beta value, average ± SD)					Gene group
						HP (-) young	*HP* (-) old	*HP* -current young	*HP* -current old	*HP*- past old	
61766	3	*PIK3CA*	409	435	107	0.007 ± 0.007	0.005 ± 0.002	0.006 ± 0.006	0.004 ± 0.007	0.012 ± 0.004	M2
72454	4	*FAT4*	387	166	5	0.010 ± 0.011	0.020 ± 0.017	0.333 ± 0.039	0.330 ± 0.196	0.199 ± 0.077	C2
72450	4	*FAT4**	387	166	5	0.013 ± 0.008	0.047 ± 0.013	0.473 ± 0.032	0.283 ± 0.274	0.269 ± 0.046	D2
132400	8	*RIMS2*	293	85	0	0.017 ± 0.013	0.062 ± 0.036	0.275 ± 0.077	0.670 ± 0.106	0.307 ± 0.148	B2
124914	8	*CSMD1*	260	7	0	0.103 ± 0.023	0.168 ± 0.025	0.598 ± 0.108	0.615 ± 0.098	0.431 ± 0.055	C2
51498	3	*CTNNB1*	253	4819	92	0.098 ± 0.031	0.097 ± 0.020	0.083 ± 0.021	0.096 ± 0.022	0.121 ± 0.025	M2
83009	5	*APC*	250	255	248	0.019 ± 0.012	0.028 ± 0.019	0.015 ± 0.012	0.015 ± 0.016	0.035 ± 0.005	M2
50643	3	*TGFBR2*	221	2705	77	0.015 ± 0.008	0.018 ± 0.010	0.006 ± 0.009	0.009 ± 0.013	0.022 ± 0.008	M2
9038	1	*MACF1*	206	577	0	0.054 ± 0.011	0.073 ± 0.036	0.107 ± 0.057	0.156 ± 0.160	0.111 ± 0.019	M2
220692	16	*CDH1*	204	12830	317	0.056 ± 0.013	0.107 ± 0.021	0.578 ± 0.091	0.386 ± 0.303	0.297 ± 0.050	D2
75704	4	*FAT1*	196	4445	2	0.076 ± 0.021	0.124 ± 0.039	0.657 ± 0.158	0.432 ± 0.255	0.422 ± 0.053	D2
116949	7	*TRRAP*	189	396	0	0.006 ± 0.007	0.013 ± 0.013	0.004 ± 0.007	0.006 ± 0.010	0.060 ± 0.015	M2
260128	20	*PTPRT*	175	5	4	0.062 ± 0.029	0.095 ± 0.040	0.727 ± 0.148	0.684 ± 0.156	0.422 ± 0.066	D2
44230	2	*ERBB4*	173	26	10	0.024 ± 0.015	0.057 ± 0.018	0.502 ± 0.156	0.511 ± 0.232	0.197 ± 0.077	C2
21060	1	*CACNA1E*	168	21	0	0.037 ± 0.013	0.102 ± 0.024	0.258 ± 0.041	0.639 ± 0.074	0.377 ± 0.073	B2
73715	4	*FBXW7*	155	442	10	0.027 ± 0.014	0.036 ± 0.023	0.012 ± 0.014	0.023 ± 0.021	0.049 ± 0.011	M2
21480	1	*HMCN1*	149	118	1	0.043 ± 0.019	0.075 ± 0.019	0.490 ± 0.065	0.330 ± 0.260	0.297 ± 0.047	D2
131940	8	*VPS13B*	139	369	1	0.010 ± 0.011	0.016 ± 0.016	0.005 ± 0.010	0.007 ± 0.011	0.026 ± 0.006	M2
127221	8	*ADRA1A*	137	31	0	0.089 ± 0.036	0.182 ± 0.026	0.495 ± 0.044	0.628 ± 0.026	0.403 ± 0.068	C2
196285	14	*NIN*	133	135	5	0.025 ± 0.007	0.027 ± 0.023	0.030 ± 0.010	0.019 ± 0.011	0.033 ± 0.010	M2
140196	9	*ASTN2*	133	69	0	0.028 ± 0.015	0.033 ± 0.014	0.096 ± 0.038	0.237 ± 0.157	0.125 ± 0.057	B2
251279	19	*RYR1*	133	3	1	0.033 ± 0.016	0.061 ± 0.030	0.031 ± 0.029	0.246 ± 0.122	0.113 ± 0.049	A2
80420	5	*MAST4*	132	372	0	0.010 ± 0.005	0.020 ± 0.012	0.012 ± 0.013	0.034 ± 0.037	0.030 ± 0.013	M2
48543	3	*ITPR1*	132	276	0	0.008 ± 0.007	0.013 ± 0.009	0.007 ± 0.007	0.006 ± 0.007	0.014 ± 0.004	M2
115919	7	*PCLO*	132	270	0	0.027 ± 0.015	0.045 ± 0.006	0.546 ± 0.104	0.327 ± 0.312	0.281 ± 0.072	D2
188842	13	*BRCA2*	129	213	75	0.020 ± 0.014	0.026 ± 0.020	0.008 ± 0.013	0.023 ± 0.019	0.040 ± 0.009	M2
204354	15	*RYR3*	129	63	0	0.013 ± 0.012	0.054 ± 0.022	0.211 ± 0.013	0.433 ± 0.038	0.206 ± 0.060	B2
116295	7	*AKAP9*	128	1394	1	0.012 ± 0.008	0.022 ± 0.018	0.010 ± 0.014	0.011 ± 0.013	0.028 ± 0.007	M2
154832	10	*FGFR2*	124	458	73	0.048 ± 0.012	0.063 ± 0.015	0.276 ± 0.048	0.218 ± 0.258	0.163 ± 0.048	D2
34190	2	*DYSF*	124	247	0	0.039 ± 0.016	0.058 ± 0.018	0.407 ± 0.030	0.254 ± 0.242	0.245 ± 0.045	D2
179374	12	*ERBB3*	123	4185	16	0.018 ± 0.009	0.028 ± 0.019	0.058 ± 0.026	0.115 ± 0.168	0.063 ± 0.021	M2

* TSS200 region identified as major in the stomach in a previous study (Yoshida S *et al.* Gastric Cancer 2016; 20:136-145.).

† Cosmic database

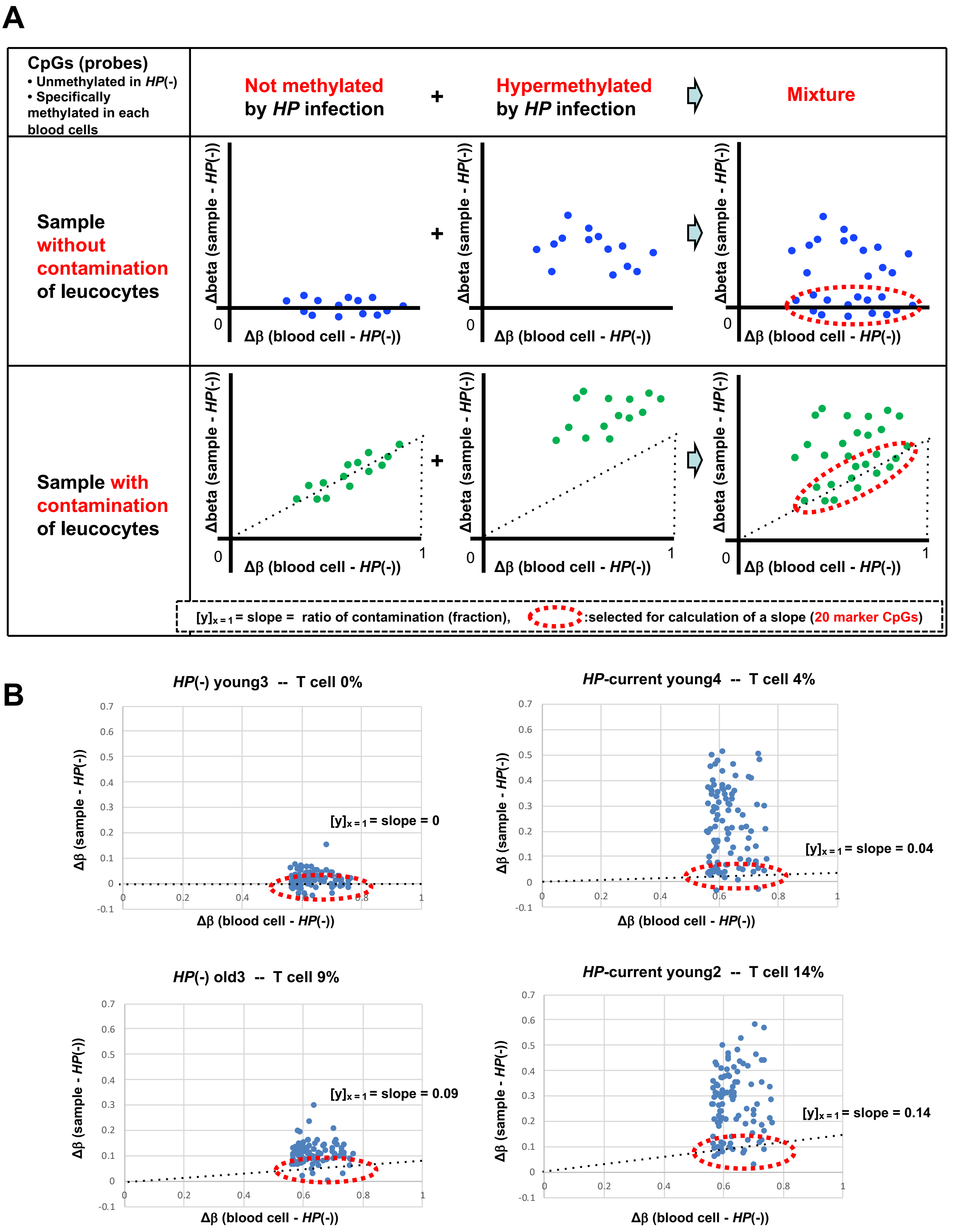

Supple Fig. 1 Yamashita *et al.*

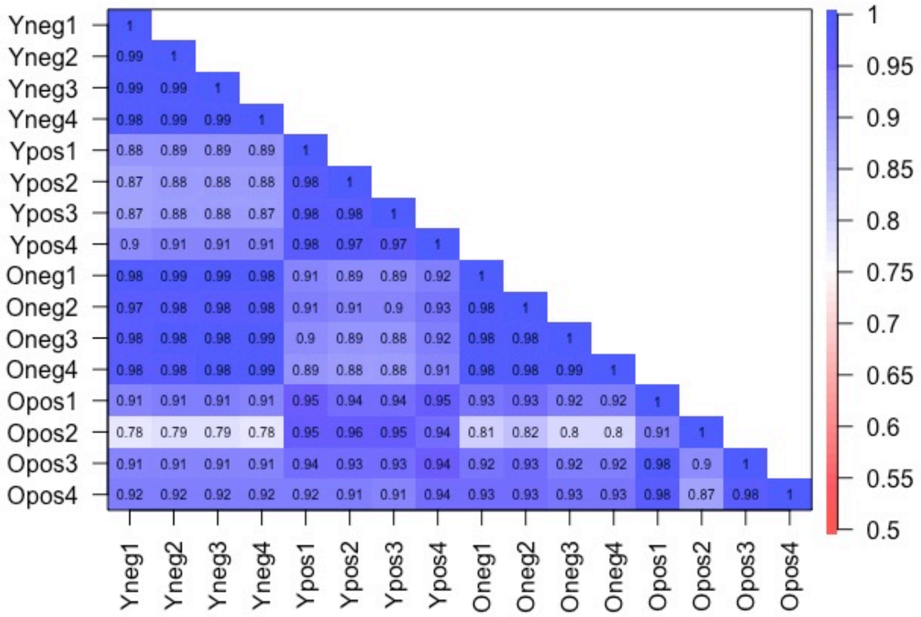

Supple Fig. 2 Yamashita *et al.*

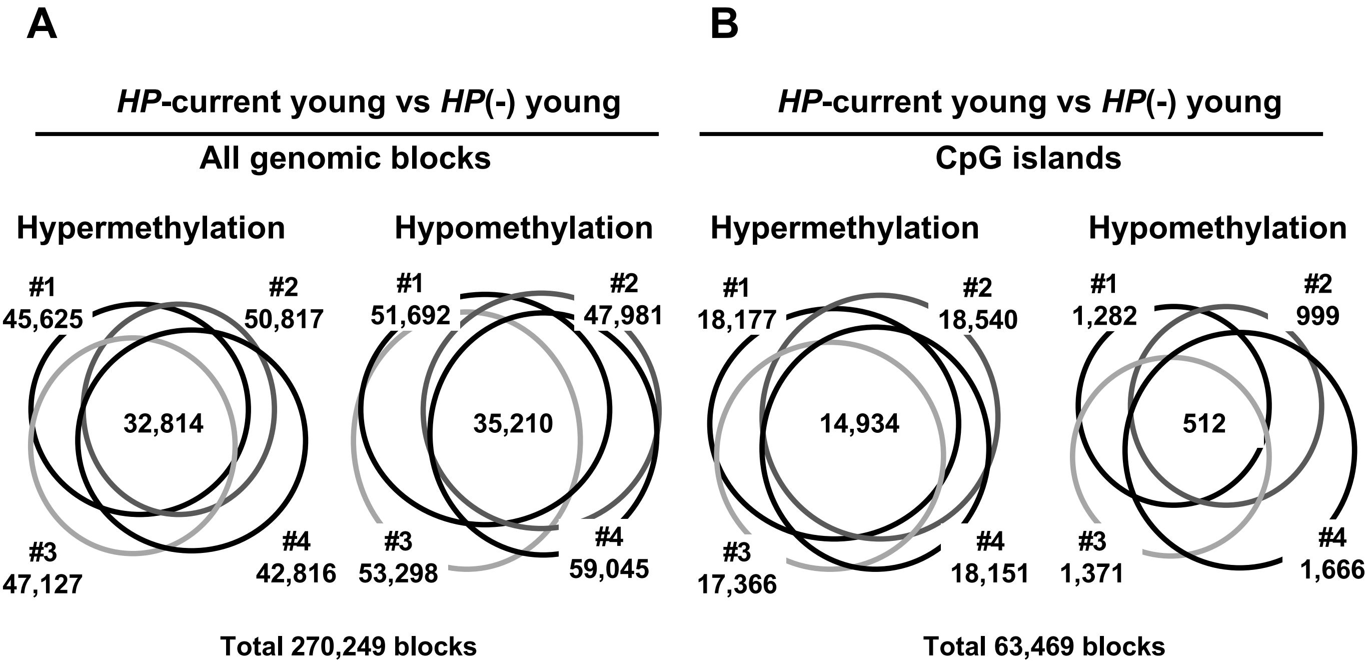

Supple Fig. 3 Yamashita *et al*.

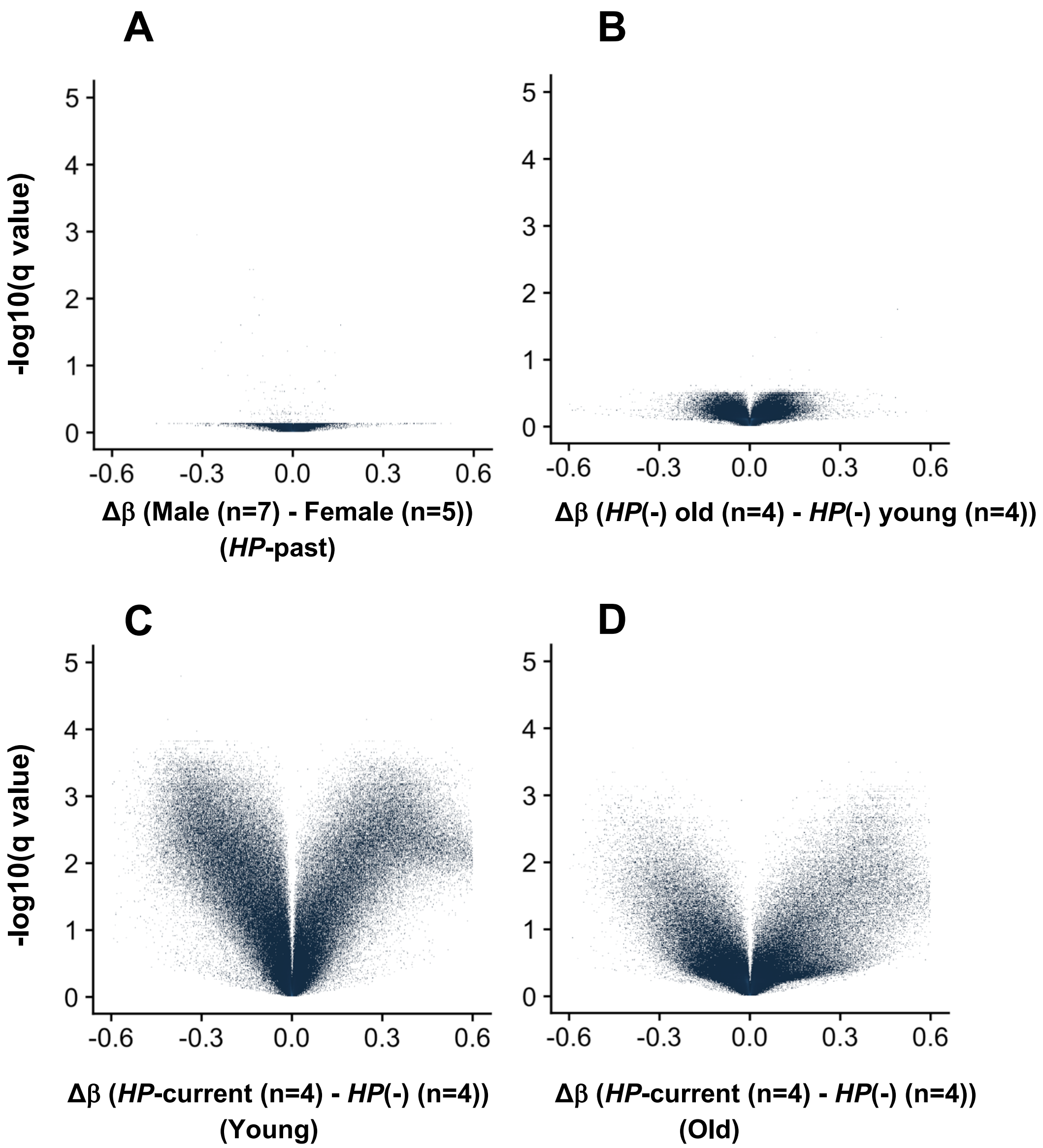

Supple Fig. 4 Yamashita *et al*.

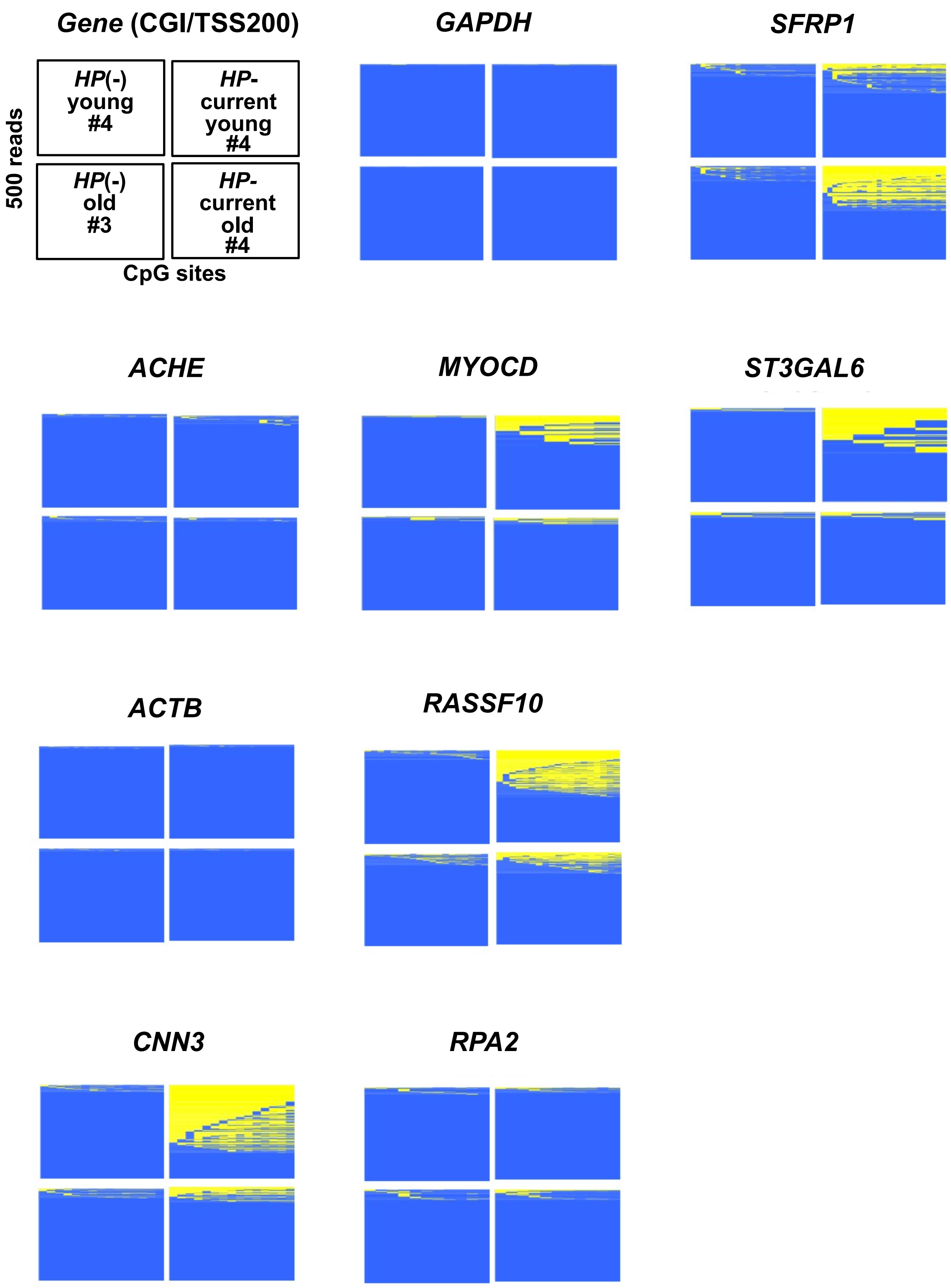

Supple Fig. 5 Yamashita *et al.*

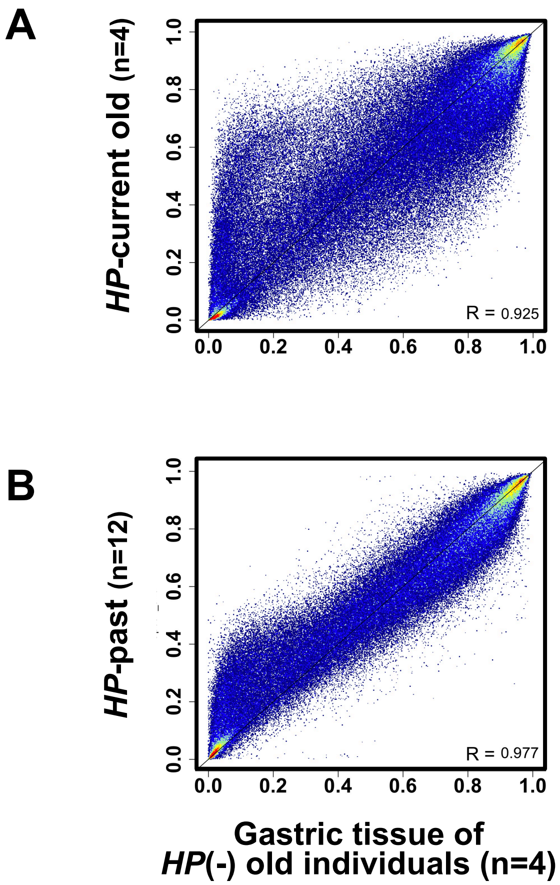

Supple Fig. 6 Nanjo *et al*.

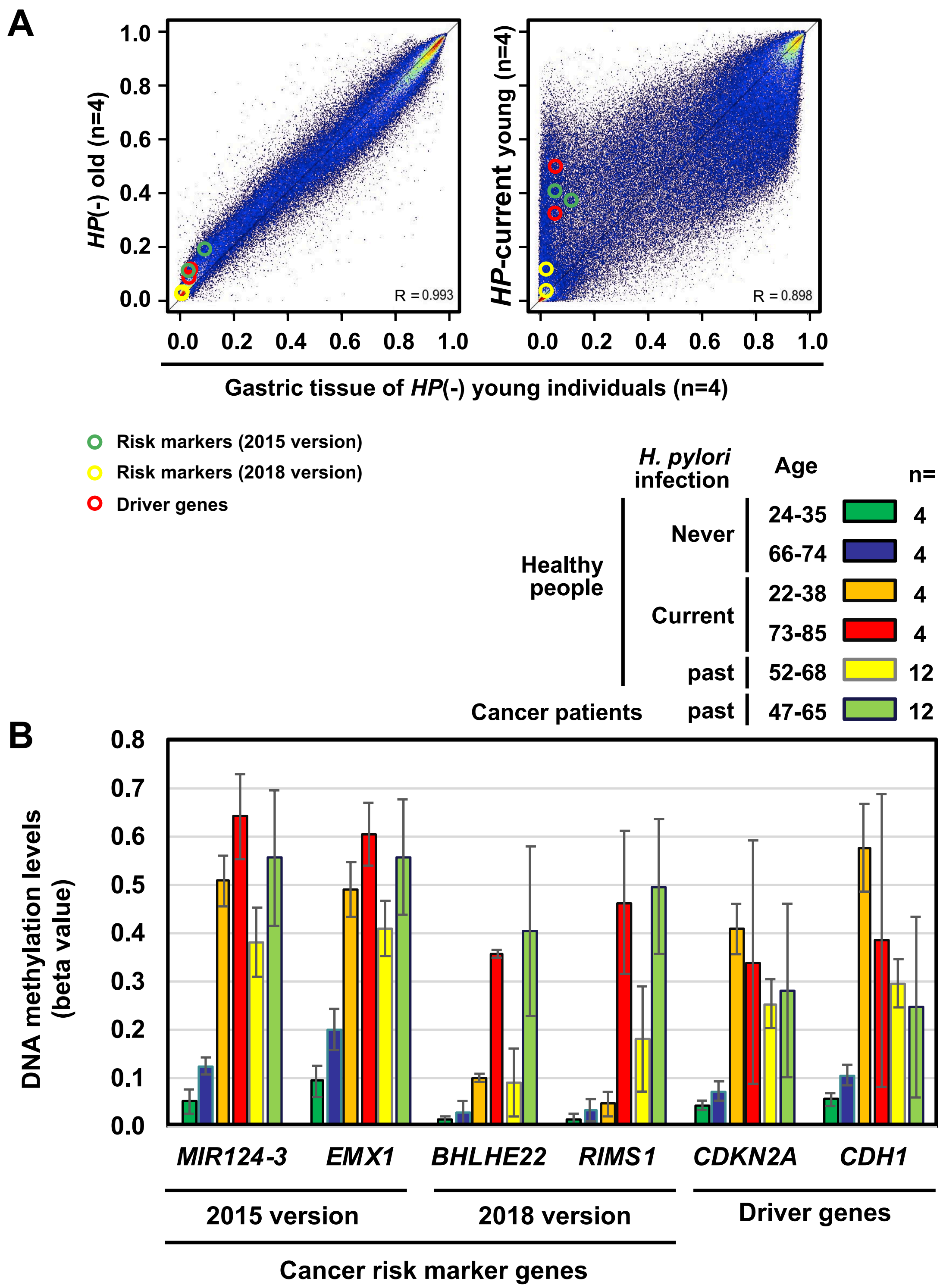

Supple Fig. 7 Yamashita *et al.*

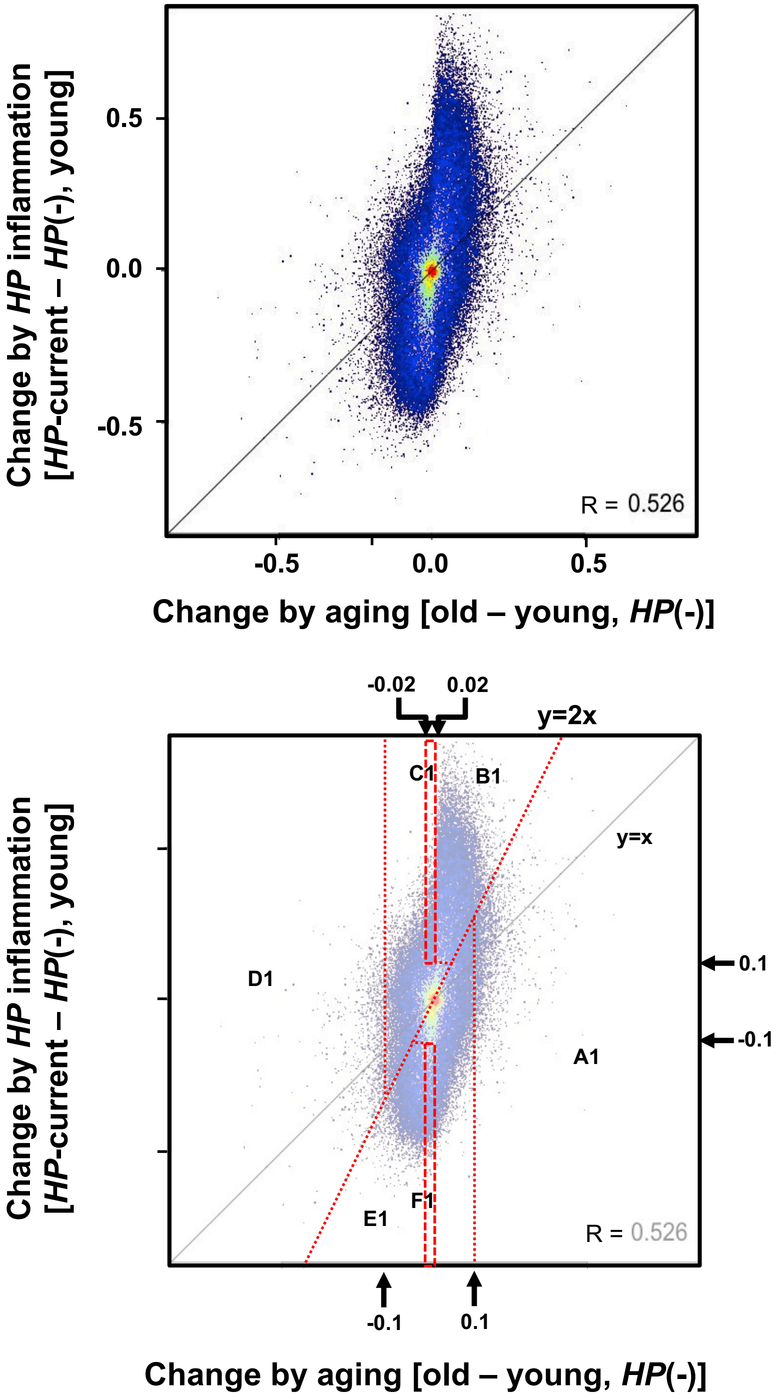

Supple Fig. 8 Yamashita *et al*.

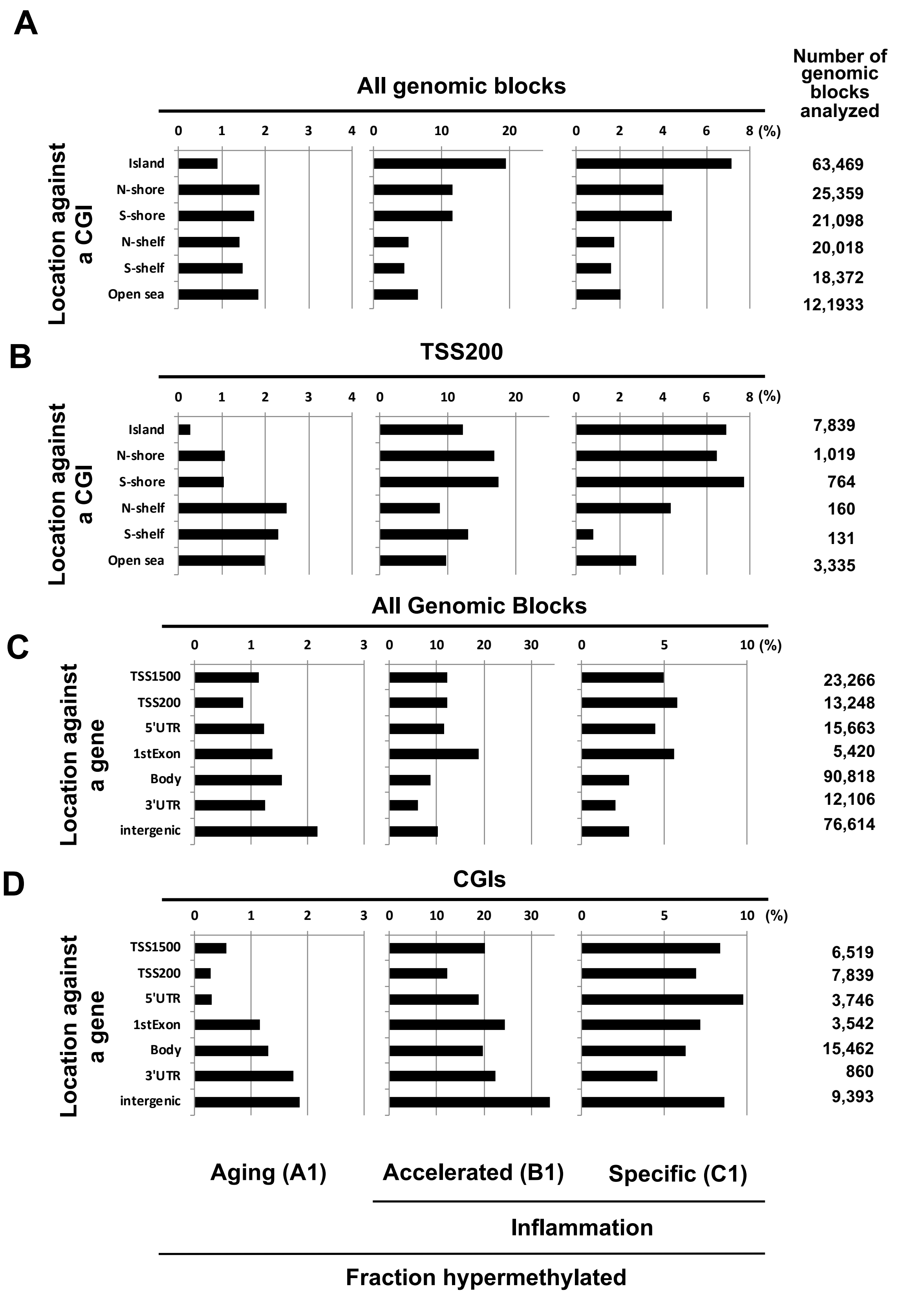

Supple Fig. 9 Yamashita *et al.*

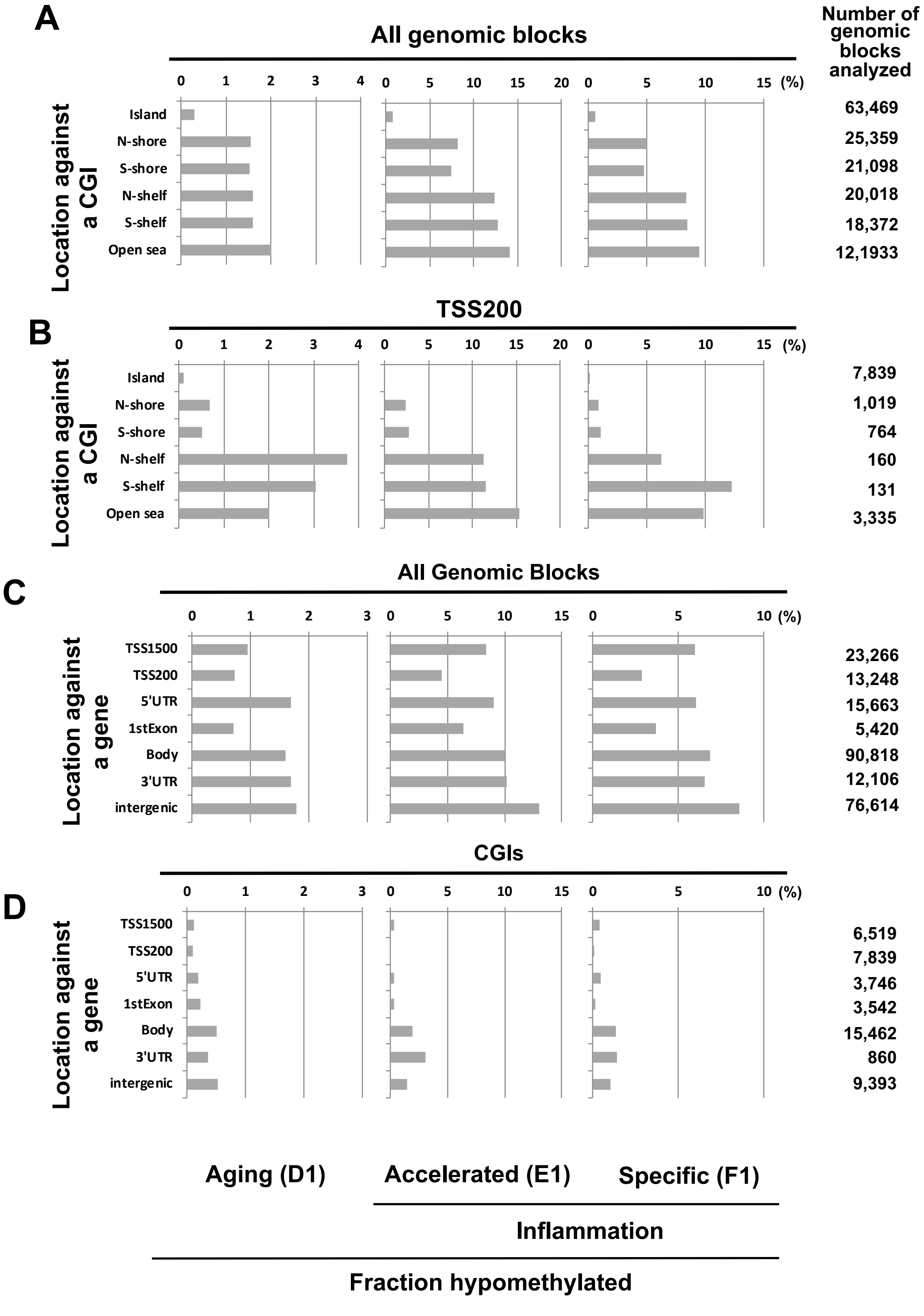

Supple Fig. 10 Yamashita *et al*.

A

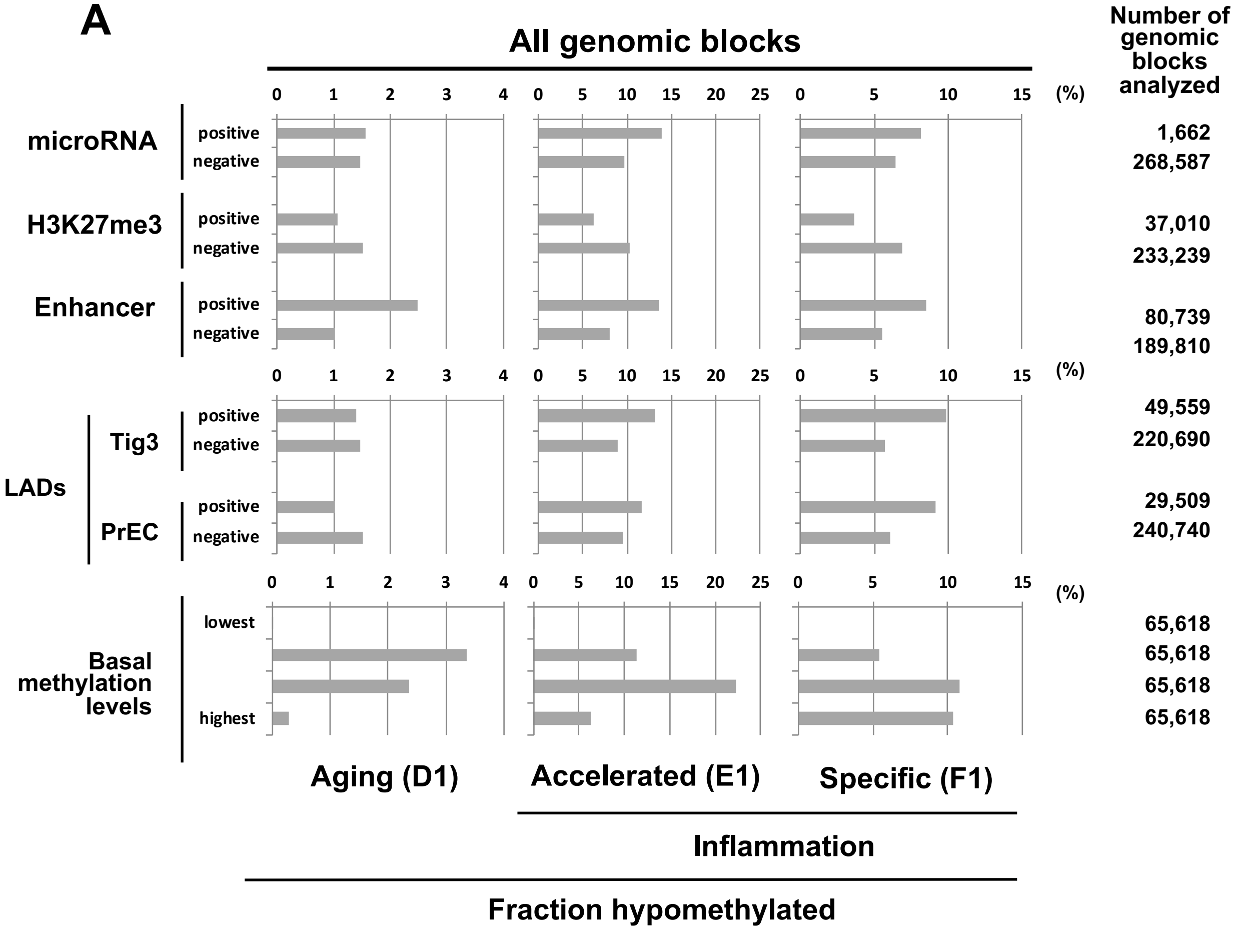

B

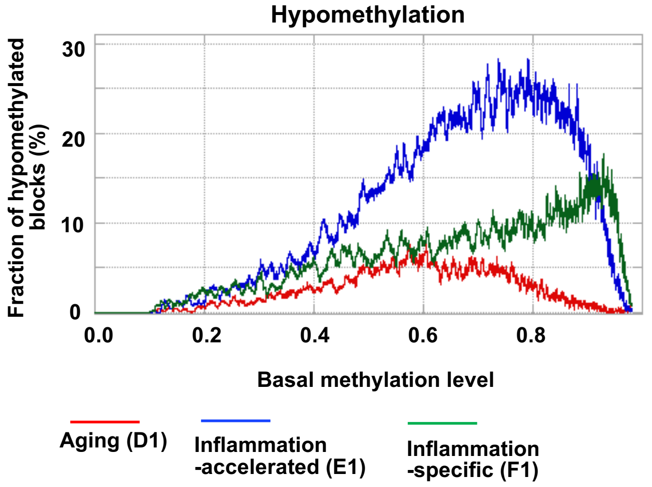

C Multivariate analysis for all genomic blocks

		Aging (D1)	Inflammation	
			Accelerated (E1)	Specific (F1)
		OR (95%CI)	OR (95%CI)	OR (95%CI)
Location against a gene	TSS1500	**0.69 (0.60-0.81)**	0.97 (0.92-1.02)	**1.17 (1.10-1.25)**
	TSS200	**0.65 (0.53-0.81)**	**0.73 (0.67-0.79)**	0.92 (0.82-1.03)
	5'UTR	1.04 (0.91-1.19)	**0.82 (0.77-0.87)**	**0.87 (0.81-0.94)**
	1st Exon	0.83 (0.60-1.16)	**1.17 (1.04-1.32)**	1.00 (0.86-1.16)
	Body	1.00 (0.93-1.08)	**0.81 (0.79-0.84)**	**0.80 (0.77-0.83)**
	3'UTR	**1.27 (1.09-1.48)**	**0.78 (0.73-0.83)**	**0.66 (0.61-0.71)**
	intergenic	1.00 (Reference)	1.00 (Reference)	1.00 (Reference)
Location against a CGI	CGI	**<u>0.16</u> (0.14-0.19)**	**<u>0.07</u> (0.07-0.08)**	**<u>0.13</u> (0.12-0.15)**
	N-shore	0.97 (0.86-1.11)	**0.67 (0.63-0.70)**	**0.68 (0.64-0.70)**
	S-shore	0.96 (0.84-1.10)	**0.59 (0.55-0.62)**	**0.65 (0.60-0.70)**
	N-shelf	1.14 (0.99-1.29)	0.99 (0.94-1.04)	0.95 (0.90-1.01)
	S-shelf	**1.16 (1.02-1.33)**	1.03 (0.98-1.08)	0.97 (0.91-1.03)
	Open sea	1.00 (Reference)	1.00 (Reference)	1.00 (Reference)
microRNA		**1.80 (1.09-2.98)**	**1.71 (1.40-2.08)**	1.05 (0.81-1.37)
Absence of H3K27me3		**1.33 (1.18-1.49)**	**1.08 (1.02-1.13)**	0.98 (0.92-1.05)
Enhancer		**1.89 (1.74-2.05)**	**1.28 (1.23-1.32)**	**1.20 (1.16-1.25)**
LAD (Tig3)		**0.87 (0.79-0.95)**	**1.32 (1.28-1.36)**	**1.56 (1.50-1.62)**
Basal methylation level		**0.70 (0.67-0.72)**	**1.03 (1.01-1.04)**	**1.55 (1.52-1.58)**

Supple Fig. 11 Yamashita *et al.*

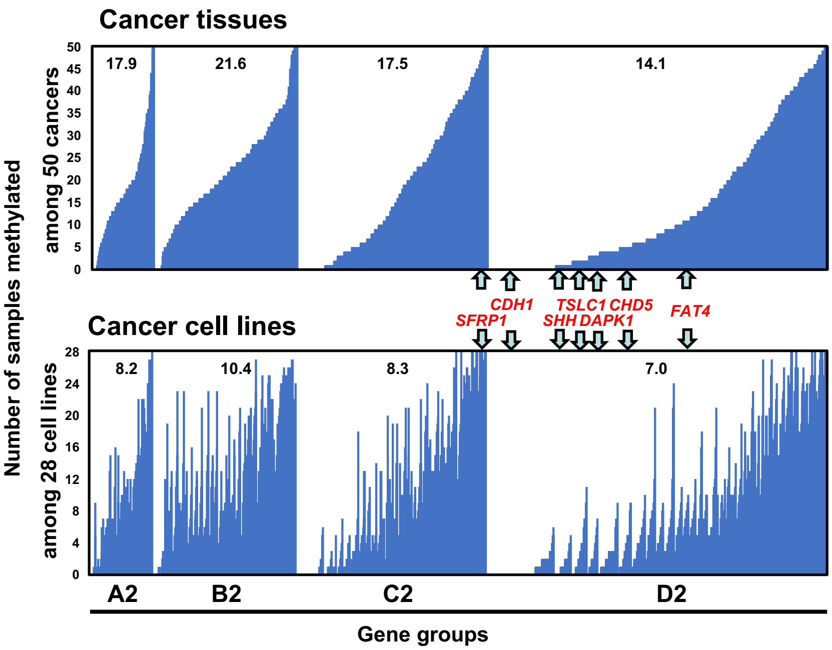

Supple Fig. 12 Yamashita *et al*.

Supplementary Figure Legends

Supplementary Fig. 1

Estimation of leucocyte fraction using DNA methylation. (A) Principle of the method for the estimation. All the marker CpG sites for T cell and NK cells, B cell, monocyte, and pan-leucocytes were screened as (1) highly methylated (β values ≥ 0.5) in a specific type of leucocytes, and (2) not methylated (β values ≤ 0.1) in the other types of leucocytes or in *HP*(-) young gastric mucosa. Here, methylation levels of the 100 candidate marker CpG sites were plotted [x = β value (blood cell - *HP*(-) young), y = β value (sample - *HP*(-) young)]. The x value of a CpG site not methylated by *HP*-triggered inflammation (left panels) represents its methylation level in a specific leucocyte, and its y value represents the product of leucocyte fraction and its methylation level. In contrast, the y value of a CpG site methylated in gastric epithelial cells (middle panels) is composed of methylation due to leucocyte contamination and in epithelial cells. Therefore, from the 100 candidates, the 20 least methylated CpG sites were selected (inside of red dotted lines, right panels) as those not methylated in epithelial cells, and their regression line was obtained to calculate the fraction of the leucocytes. A fraction of each group of cells in each sample was calculated as the β value of a sample when the methylation level of the leucocyte is 100% ([y]x = 1, slope of the regression line). (B) Four examples of the estimation of leucocyte fraction using DNA methylation.

Supplementary Fig. 2

Similarity of methylated CpG sites among different samples in a group. Heatmaps of correlation coefficients of methylation levels among different samples are shown. Correlation coefficients of methylation levels were very high (> 0.96) among the four samples

within a group, with an exception of the *HP*-current old#2 sample.

Supplementary Fig. 3

Overlapping of regions of aberrant DNA methylation among individuals. The Venn diagrams show the overlap of genomic blocks hyper- or hypomethylated by *HP* infection (young) using all genomic blocks (A) or blocks in CGIs (B). The consistency of differentially methylated genomic blocks was very high for hypermethylation of CpG islands, high for hyper- and hypo-methylation of all genomic blocks, and moderate for hypomethylation of CpG islands.

Supplementary Fig. 4

Statistical analysis of difference of methylation levels (β value) between two groups using volcano plot. (A) Sex difference. (B) Aging. (C) Current *HP* infection in young individuals, compared with that in *HP*(-) young individuals. (D) Current *HP* infection in old individuals, compared with that in *HP*-past (old) individuals.

Supplementary Fig. 5

Targeted deep bisulfite sequencing for the CGI/TSS200 regions of nine genes. Yellow bar; methylated CpG, Blue bar; unmethylated CpG.

Supplementary Fig. 6

Aberrant DNA methylation induced in gastric mucosa by current and past infection. (A) Global difference of methylation levels (β value) between *HP*-negative old group (*HP*(-) old) and *HP*-positive old group (*HP*-current old). (B) Global difference of methylation levels (β value) between *HP*-negative old group (*HP*(-) old) and past-infected group. Smaller

numbers of genomic blocks were hyper- and hypomethylated, respectively, by past infection in gastric mucosa.

Supplementary Fig. 7

Methylation levels of driver genes and risk marker genes for gastric carcinogenesis (CGI/TSS200). The risk marker genes version 2015 were used for cancer risk prediction in a multicentre prospective cohort study [14, 15]. The risk marker genes version 2018 were recently identified for gastric cancer risk stratification in individuals after *Helicobacter pylori* eradication [16]. (A) The driver genes and risk marker genes version 2015 showed a large methylation difference by current *HP* infection in young individuals. (B) The driver genes showed slight methylation differences between gastric mucosae from cancer patients and those from healthy individuals with past *HP* infection. In contrast, the risk marker genes, especially version 2018, showed large differences between gastric mucosae from cancer patients and those from healthy individuals with past *HP* infection.

Supplementary Fig. 8

Definition of genomic blocks differentially methylated by aging and by inflammation. Increases of methylation levels by aging and those by inflammation were plotted. Six areas (A1 to F1) were defined as age-related, inflammation-accelerated, and inflammation-specific hyper- and hypomethylation.

Supplementary Fig. 9

Influence of genomic regions, classified by relative locations against a CGI or a gene, on hypermethylation. Influence on hypermethylation was estimated by the fraction of hypermethylated genomic blocks. (A) Influence of relative location against a CGI on

hypermethylation for all genomic blocks. (B) For blocks in TSS200. (C) Influence of relative location against a gene on hypermethylation classified for all genomic blocks. Relative locations against a gene were defined as follows: 1stExon = within the first exon of the gene, Body = within the body of the gene beyond the first exon, 5'UTR = in the 5' untranslated region of the gene, 3'UTR = in the 3' UTR of the gene, TSS200 = within 200bp upstream of the transcriptional start site, TSS1500 = between 200-1500 bp upstream of the TSS. (D) For genomic blocks in CGIs.

Supplementary Fig. 10

Influence of genomic regions, classified by relative location against a CGI or a gene, on hypomethylation. Influence on hypomethylation was estimated by the fraction of hypomethylated genomic blocks. (A) Influence of relative location against a CGI on hypomethylation for all genomic blocks. (B) For blocks in TSS200. (C) Influence of relative location against a gene on hypomethylation for all genomic blocks. (D) For genomic blocks in CGIs.

Supplementary Fig. 11

Influence of microRNA, H3K27me3, enhancers, LADs, and basal methylation levels, in addition to genomic factors, on hypomethylation. (A) Univariate analysis. The influence of each factor on hypomethylation was estimated by the fraction of hypomethylated genomic blocks. (B) Influence of basal methylation levels on hypomethylation. The fraction of hypomethylated genomic blocks among the blocks with the same basal methylation levels in *HP*(-) young gastric mucosa was plotted. Basal methylation levels smaller than 0.9 of β value were essential for hypomethylation by aging. In contrast, hypomethylation by inflammation was induced even in genomic blocks with basal methylation levels larger than

0.9, and was very frequent with basal methylation levels between 0.6 and 0.9. (C) Multivariate analysis involving locations against a gene and a CGI, microRNA, H3K27me3, enhancers, LADs, and basal expression levels. Boldface; statistically significant, Underline; odds ratio >2.0 or <0.5.

Supplementary Fig. 12

Methylation frequency of promoter CGIs in areas A2-D2 in 50 primary gastric cancers and 28 gastric cancer cell lines. The data of methylation levels were obtained from our previous study [Yoda Y *et al*. Gastric Cancer. 2015;18:65–76.], and genomic blocks whose β values > 0.4 in cancer tissues or > 0.8 in cancer cell lines were considered as methylated. The number shown in each group is a mean of number of samples with methylation. Methylation frequency was especially low for CGI/TSS200 in area D2. Driver genes known to be methylated in gastric cancer are enriched in area D2.
